# Effect of Intramuscular vs Intra-articular Glucocorticoid Injection on Pain Among Adults With Knee Osteoarthritis: The KIS Randomized Clinical Trial

**DOI:** 10.1001/jamanetworkopen.2022.4852

**Published:** 2022-04-05

**Authors:** Qiuke Wang, Marianne F. Mol, P. Koen Bos, Desirée M. J. Dorleijn, Marijn Vis, Jacobijn Gussekloo, Patrick J. E. Bindels, Jos Runhaar, Sita M. A. Bierma-Zeinstra

**Affiliations:** 1 Department of General Practice, Erasmus MC University Center Rotterdam, Rotterdam, the Netherlands; 2 Department of Orthopaedic Surgery, Erasmus MC University Center Rotterdam, Rotterdam, the Netherlands; 3 Department of Orthopaedic Surgery, Leiden University Medical Centre, Leiden, the Netherlands; 4 Department of Rheumatology, Erasmus MC University Center Rotterdam, Rotterdam, the Netherlands; 5 Department of Public Health and Primary Care, Leiden University Medical Center, Leiden, the Netherlands; 6 Section of Gerontology and Geriatrics, Department of Internal Medicine, Leiden University Medical Center, Leiden, the Netherlands

## Abstract

**Importance:**

Intra-articular (IA) glucocorticoid injection is widely used in patients with knee osteoarthritis (OA), but the safety of this technique is in question among physicians. Intramuscular (IM) glucocorticoid injection could be an alternative approach.

**Objective:**

To investigate whether an IM glucocorticoid injection is noninferior to an IA glucocorticoid injection in reducing knee pain for patients with knee OA in primary care.

**Design, Setting, and Participants:**

The KIS trial, a multicenter, open-label, randomized clinical noninferiority trial including patients with symptomatic knee OA, was conducted in 80 primary care general practices in the southwest of the Netherlands. The study was conducted from March 1, 2018, to July 28, 2020.

**Interventions:**

Patients were randomly allocated to receive an injection of triamcinolone acetonide, 40 mg, either IM in the ipsilateral ventrogluteal region or IA in the knee joint. All patients were followed up for 24 weeks.

**Main Outcomes and Measures:**

The pain score at 4 weeks measured with Knee Injury and Osteoarthritis Outcome Score (range, 0-100; 0 indicates extreme pain), with a noninferiority margin of −7 (IM minus IA). A per-protocol analysis was prespecified as the primary analysis.

**Results:**

A total of 145 patients (94 women [65%]; mean [SD] age, 67 [10] years) were included; of these, 138 patients (IM, 72; IA, 66) were included in the per-protocol analysis. Clinically relevant improvements in knee pain were reached up to 12 weeks after the injection in both groups. At 4 weeks, the estimated mean difference in the Knee Injury and Osteoarthritis Outcome Score between the 2 groups was −3.4 (95% CI, −10.1 to 3.3). Noninferiority could not be declared because the lower limit exceeded the noninferiority margin. Intramuscular injection was noninferior to IA injection at 8 (mean difference, 0.7; 95% CI, −6.5 to 7.8) and 24 (mean difference, 1.6; 95% CI, −5.7 to 9.0) weeks. No significant difference was found among all the secondary outcomes. These results were similar for the sensitivity analysis in an intention-to-treat population. The most frequently reported adverse events were hot flush (IM, 7 [10%] vs IA, 14 [21%]) and headache (IM, 10 [14%] vs IA, 12 [18%]), and all events were classified as nonserious.

**Conclusions and Relevance:**

Based on the findings of this trial, among patients with knee OA in primary care, IM glucocorticoid injection could present an inferior effect in reducing pain at 4 weeks compared with IA injection. Noninferiority of an IM injection was observed at 8 and 24 weeks after injection. This trial provides data for shared decision-making, taking into account the advantages and disadvantages of both types of injections.

**Trial Registration:**

Dutch Trial Registry: NTR6968

## Key Points

**Question:**

Is an intramuscular glucocorticoid injection noninferior to an intra-articular glucocorticoid injection in reducing knee pain in patients with knee osteoarthritis in primary care?

**Findings:**

This randomized clinical trial including 145 patients with symptomatic knee osteoarthritis found intramuscular injection of the glucocorticoid triamcinolone acetonide could present an inferior effect in reducing pain at 4 weeks compared with the intra-articular injection. Noninferiority of an intramuscular injection was observed at 8 and 24 weeks after injection.

**Meaning:**

The findings of this trial suggest both types of injection should be considered effective strategies and that a shared decision-making process should take place between clinicians and patients with knee osteoarthritis when a glucocorticoid injection is indicated.

## Teaser

This randomized clinical trial compares the effect of intramuscular vs intra-articular injection of a glucocorticoid on pain in patients with symptomatic knee osteoarthritis.

## Introduction

Osteoarthritis (OA) is a leading cause of disability, and the knee is the most commonly affected joint.^1^ Key treatment strategies, such as disease education, exercise therapy, and weight loss, in combination with pain medications are usually indicated for patients with knee OA in clinical practice. Among the pain medications, intra-articular (IA) glucocorticoid injection is one of the most widely used.^2^ Clinical trials have demonstrated the short-term effectiveness of IA glucocorticoid injection in reducing moderate to severe knee pain.^3,4,5^ Several professional guidelines recommend use of IA glucocorticoid injection for patients with knee OA who have not responded to oral or topical analgesics.^6,7,8,9^

However, the safety of injecting a glucocorticoid into the knee is increasingly drawing concerns among physicians.^10^ A 2-year randomized clinical trial showed IA injection of glucocorticoids would result in significantly greater cartilage loss.^11^ In addition, although rare, IA injection is associated with higher risks of septic arthritis and postoperative joint infection,^12^ for which a 3-month minimum interval between injection and further operations is recommended.^10^ Another obstacle of implementing IA injection is that, in primary care, general practitioners (GPs) may not feel competent to place the needle into the knee joint.^13^

Intramuscular (IM) injection could be an alternative approach for glucocorticoid administration in patients with knee OA because it eliminates the direct risks of toxic effects on cartilage and septic arthritis and is easier to perform than IA injection. Intramuscular glucocorticoid injection has been reported to be beneficial in relieving pain in other musculoskeletal diseases, such as rotator cuff disease^14^ and hand OA.^15^ In a double-blind trial conducted by our team, IM glucocorticoid injection was superior to placebo injection for reducing pain in patients with hip OA up to at least 12 weeks.^16^ To our knowledge, no study has evaluated the analgesic effect of IM glucocorticoid injection for knee OA or directly compared its effect with IA glucocorticoid injection.

Therefore, we performed the KIS randomized clinical trial to assess the effectiveness of IM glucocorticoid injection in patients with symptomatic knee OA in primary care, compared with standard IA glucocorticoid injection. The primary objective was to investigate whether an IM injection is noninferior to the IA injection in reducing knee pain at 4 weeks after injection.

## Methods

### Study Design

KIS was a multicenter, open-labeled, parallel, noninferiority randomized clinical trial with a follow-up period of 24 weeks. A detailed study protocol has been published.^17^ The trial protocol and statistical analysis plan as approved are available in Supplement 1. The medical ethics committee of Erasmus University Medical Center approved the protocol (MEC 2017-563), and all included patients provided written informed consent before baseline measures were obtained; participants did not receive financial compensation. Reporting the results followed the Consolidated Standards of Reporting Trials (CONSORT) reporting guideline for noninferiority and equivalence trials.^18^

### Patients

We recruited patients between March 1, 2018, and February 11, 2020, at 80 general practices in the southwest region of the Netherlands. Final follow-up of the study was July 28, 2020. Inclusion criteria included age 45 years and older, consulted in primary care for knee symptoms during the past 5 years with knee OA diagnosed by a GP, presence of symptomatic knee OA for at least 3 months before enrollment, and moderate to severe knee pain over the past week (numeric rating scale score ≥3 on a scale of 0-10; 0 indicates no pain).^16,17^ The treating GP assessed whether there was an indication for a glucocorticoid injection for the eligible patients. The National GP guideline recommends glucocorticoid injection for patients with knee OA who have a flare of knee pain and/or do not respond to other pain medications.^19^ For patients with bilateral knee OA, the most painful knee was chosen as the index knee.

We excluded patients if they were using oral glucocorticoids, had received IA injection of glucocorticoids within the past 6 months, were allergic to glucocorticoids, had a local or systemic infection or recent vaccination with live attenuated vaccine, had type 1 or poorly controlled type 2 diabetes (assessed by the GP), had inflammatory rheumatic diseases (eg, rheumatoid arthritis, psoriatic arthritis, and spondyloarthropathies), had coagulopathy (or were receiving anticoagulants), had a history of or current gastric or duodenal ulcer, had contacted an orthopedic surgeon for potential surgical management of the knee, or were incapable of completing questionnaires in Dutch or giving informed consent.

### Interventions

Patients in the IM group received a single intramuscular injection of triamcinolone acetonide, 40 mg (1 mL), in the ipsilateral ventrogluteal region. Patients in the IA group received a single standard IA injection (superolateral approach) of triamcinolone acetonide, 40 mg (1 mL), in the index knee.^17^ The treating GP prepared and administered all the injections within 1 week after completion of the baseline assessment. The GPs were instructed not to use local anesthetics during the injection and were offered an opportunity of IA injection training under supervision of an experienced orthopedic surgeon (P.K.B.).

### Randomization and Blinding

An independent data manager, who was not involved in the clinical procedure, prepared a computer generalized randomization list using 1:1 allocation. To ensure concealment of allocation, random blocks of 8, 6, or 4 were used and the digital randomization list was kept on an encrypted website. After the patient had provided written informed consent and finished baseline assessments, a member of the research team performed the randomization on the encrypted website and then informed the patient and GP of the randomization result. The researchers involved in data analysis were blinded to the treatment allocation and were required to write a concrete analysis plan before conducting the data analysis.

### Outcomes

We measured outcomes at baseline (the day of completing baseline assessments), and 2, 4, 8, 12, and 24 weeks after administration of the injection via digital or paper questionnaires. Two of us (Q.W. and P.K.B.) did the baseline Kellgren and Lawrence grade scoring blinded to the allocated treatment (interrater reliability: prevalence-adjusted κ value: 0.84; 95% CI, 0.75-0.93). A consensus meeting was held for making final decisions for discrepant grades. The primary outcome was the severity of knee pain at 4 weeks measured with the Knee Injury and Osteoarthritis Outcome Score (KOOS) pain subscale (0-100; 0 indicates extreme pain).

Secondary outcomes included the KOOS pain score at 2, 8, 12, and 24 weeks; KOOS symptom, function, sport and recreation, and quality-of-life scores (0-100; 0 indicates extreme symptoms); Western Ontario and McMaster Universities Osteoarthritis Index pain, function, stiffness, and total scores (0-100; 0 indicates no pain); numeric rating scale of knee pain during the past week (0-10; 0 indicates no pain); Intermittent and Constant Osteoarthritis Pain (0-100; 0 indicates no pain); and EuroQol with 5 dimensions and 5 response levels (−0.446 to 1.000; −0.446 indicates worst health-related quality of life).^20^ In addition, we measured perceived recovery and the percentage of responders defined by the OMERACT-OARSI criteria.^21^ Patients’ perceived recovery was measured with a 7-point Likert scale, and results were dichotomized into recovered (complete recovery, much improved, and slightly improved) or not recovered (no change, slightly worse, much worse, and worse than ever). The OMERACT-OARSI responders are those with high improvements (≥50% and absolute increase ≥20 points from baseline) in KOOS pain or function score; if that level is not achieved, then improvement in at least 2 of the 3 following domains: greater than or equal to 20% and greater than or equal to 10-point improvement in the KOOS pain score, greater than or equal to 20% and greater than or equal to 10-point improvement in the KOOS function score, and patient-perceived recovery in global assessment (complete recovery, much improved, and slightly improved). We measured adverse events at 2 weeks’ follow-up and asked patients about cointerventions (oral medication, additional IA injection, and visit to medical care professional) at all follow-up times.

### Sample Size

This study was powered to test whether IM injection was noninferior to IA injection regarding the KOOS pain score at 4 weeks’ follow-up. We used data from the trial of Henriksen et al^22^ for estimating an SD of 16 for the KOOS pain score. The minimal clinically important difference for KOOS pain score is 9.^23^ We prespecified the noninferiority margin at 7, which is slightly smaller than the minimal clinically important difference. A sample size of 130 patients (65 per group) was needed to achieve 80% power at a significance level of α = .05 to detect noninferiority, assuming the true between-group mean (SD) was 7 (16). Based on our prior experience in glucocorticoid injection trials, we expected a low rate of loss to follow-up of 5%.^16,24^ Therefore, we aimed to recruit 137 patients.

### Statistical Analysis

As recommended for noninferiority trials, we conducted data analysis primarily on the prespecified per-protocol principle.^17,18,25^ We included patients who received allocated injections into these analyses. Patients in the IM group who received an additional IA injection within 8 weeks were excluded from the per-protocol analysis.^17^ We repeated the analysis on the intention-to-treat (as randomized) principle for sensitivity analysis, which included all patients and grouped patients according to randomization.

We describe baseline characteristics as mean (SD) or number (percentage) as appropriate. We used linear mixed models with repeated measures to calculate mean between-group differences (calculated by estimated marginal means) and their 95% CIs over time for continuous outcomes. An unstructured covariance structure was chosen because it resulted in the lowest Akaike information criterion. We incorporated time and time by intervention group interaction into fixed effects and adjusted the analysis for the baseline value of the outcome and covariates with clinically relevant baseline differences (>10%) between the 2 groups. We calculated the effect size (Cohen *d*) by dividing estimated mean difference by the pooled SD at each follow-up. We used generalized estimating equations with repeated measures to determine odds ratios (ORs) (calculated by estimated marginal percentages) and their 95% CIs over time for the outcomes of perceived recovery and OMERACT-OARSI responder. Similarly, we adjusted these analyses for baseline KOOS pain score and the same baseline covariates as in the linear mixed models.

For the KOOS pain score, we assessed noninferiority at all time points by checking the lower limit of 2-sided 95% CIs for mean differences (IM minus IA) with a noninferiority margin of −7. Noninferiority was declared if the lower limit did not exceed the noninferiority margin. For other outcomes, we performed superiority tests only at the 2-sided .05 significance level.

We performed an explorative, predefined subgroup analysis for assessing the interaction effects of injection and baseline severity of knee pain (numeric rating scale score ≥7 vs <7) on KOOS pain scores by adding the interaction term into the linear mixed models.^17^ The amount of missing data was small (4%) and was not imputed; the above-described models take the missing values into account.^26^ All analyses were performed using SPSS, version 25.0 (IBM Corp).

## Results

### Patients

**Figure 1.**
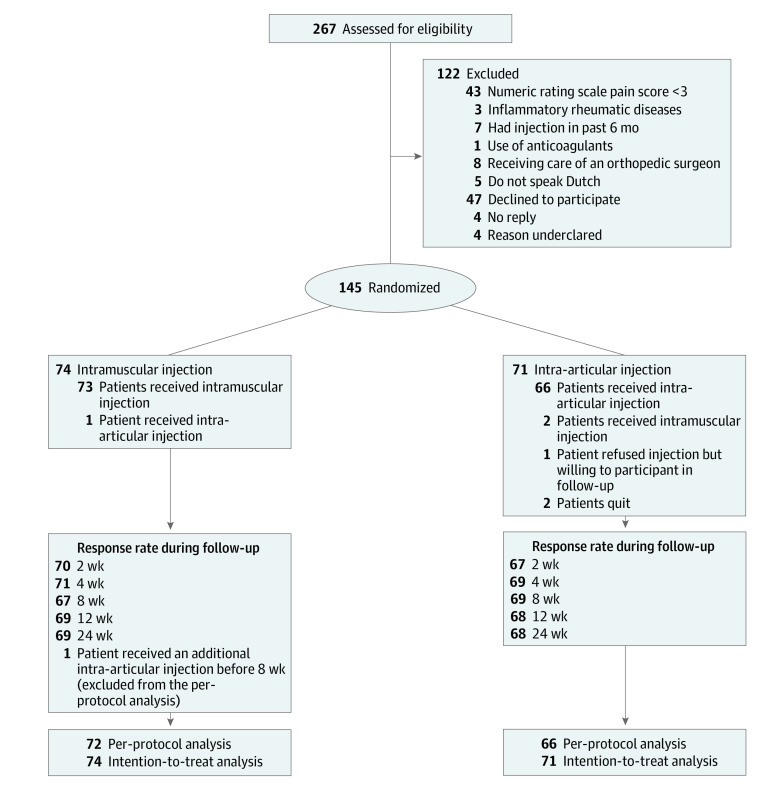
Flow of Study Participants

Between March 1, 2018, and February 11, 2020, 267 patients were assessed for eligibility; of these, 145 were randomized (94 [65%] women; 51 [35%] men; mean [SD] age, 67 [10] years) (Figure 1; Table 1). More patients reported a preference for IM than IA injection (47% vs 19%) at baseline. Of the randomized patients, 74 were allocated to the IM injection group and 71 to the IA injection group. On the injection day, based on GP reports, 1 patient in the IM group received an IA injection; in the IA group, 2 patients received IM injections and 3 patients refused injection. In addition, 1 patient in the IM group received an additional IA injection before 8 weeks’ follow-up. Therefore, 138 patients (95%) were included in the per-protocol analysis and 145 patients were included in the intention-to-treat analysis. Baseline characteristics are presented in Table 1.

**Table 1.** Baseline Characteristics of Total Participants and Participants Included in Per-Protocol Analysis

Characteristic	No. (%)		
	Total participants (n = 145)	Per-protocol analysis	
		IM injection (n = 72)	IA injection (n = 66)
Age, mean (SD), y	67 (10)	67 (11)	68 (9)
Sex			
Women	94 (65)	40 (56)	49 (74)
Men	51 (35)	32 (44)	17 (26)
BMI, mean (SD)	28.9 (5.1)	28.9 (4.5)	28.9 (5.8)
Educational level, college/university	29 (20)	21 (29)	8 (12)
Employed	53 (37)	32 (44)	19 (29)
Comorbidities			
Hip OA	21 (14)	6 (8)	13 (20)
Hand OA	44 (30)	18 (25)	23 (35)
Neck-shoulder symptom	45 (31)	23 (32)	17 (26)
Foot problem	38 (26)	14 (19)	21 (32)
Diabetes	10 (7)	4 (6)	5 (8)
Depression	9 (6)	8 (11)	1 (1)
Duration of knee OA, mean (SD), y	4.7 (4.8)	5.5 (5.6)	3.6 (3.6)
KOOS score, mean (SD)^a^			
Symptom	55.0 (17.2)	55.1 (17.6)	54.3 (16.6)
Pain	47.7 (17.1)	49.1 (17.5)	46.1 (16.4)
Function	49.9 (19.7)	52.5 (20.2)	47.0 (18.2)
Sport	16.2 (17.6)	18.0 (19.5)	12.8 (13.9)
Quality of life	33.5 (16.2)	33.4 (16.5)	33.1 (15.8)
WOMAC score, mean (SD)^b^			
Total	50.3 (18.7)	47.9 (19.2)	52.9 (17.2)
Pain	47.0 (19.6)	45.4 (20.1)	48.5 (18.7)
Function	50.1 (19.7)	47.5 (20.2)	53.0 (18.2)
Stiffness	59.2 (20.4)	56.6 (20.7)	63.1 (19.4)
NRS pain of last week, mean (SD)^c^	6.4 (1.7)	6.4 (1.8)	6.6 (1.4)
Knee OA flare			
Pain increased during the past 24 h, yes	77 (53)	34 (47)	40 (61)
Degree of stiffness during the past 24 h, mean (SD)^d^	5.6 (2.3)	5.3 (2.4)	6.1 (2.1)
Joint felt swollen during the past 24 h, yes	58 (40)	30 (42)	25 (38)
Degree of swelling, mean (SD)^e^	3.4 (3.1)	3.3 (2.9)	3.4 (3.1)
Pain pattern of last week			
Slight fluctuations	65 (45)	29 (40)	32 (49)
Persistent pain with pain attacks	25 (17)	16 (22)	9 (14)
Pain attacks but pain free in between	38 (26)	21 (29)	16 (24)
Pain attacks with pain in between	11 (8)	2 (3)	8 (12)
Other	6 (4)	4 (6)	1 (1)
Radiating pain during last week	81 (56)	46 (64)	34 (52)
ICOAP scores, mean (SD)^f^			
Total	44.3 (20.3)	44.1 (20.6)	44.8 (19.4)
Intermittent pain	46.1 (20.1)	46.5 (20.0)	46.3 (19.5)
Continuous pain	42.0 (22.9)	41.3 (22.5)	43.1 (23.2)
IPAQ category			
Inactive	31 (21)	20 (28)	9 (14)
Minimally active	25 (17)	14 (19)	9 (14)
Active	89 (61)	38 (53)	48 (73)
EQ-5D-5L, mean (SD)^g^	0.59 (0.30)	0.59 (0.31)	0.59 (0.30)
Medication use			
Acetaminophen	40 (28)	16 (22)	23 (35)
NSAID	27 (19)	14 (19)	11 (17)
Opiate	5 (3)	2 (3)	2 (3)
Participant’s preference on injection site^h^			
IA	27 (19)	13 (18)	11 (17)
IM	68 (47)	33 (46)	33 (50)
No preference	49 (34)	26 (36)	21 (32)
Participants’ expected effects of injection, much/very much improved	101 (70)	47 (65)	49 (74)
ACR clinical OA^i^	120 (83)	58 (81)	58 (88)
Tibiofemoral joint Kellgren and Lawrence grade, No./total No. (%)^j^			
Grade 1	11/131 (8)	9/65 (14)	2/62 (3)
Grade 2	56/131 (43)	22/65 (34)	33/62 (53)
Grade 3	53/131 (40)	27/65 (41)	24/62 (39)
Grade 4	11/131 (8)	7/65 (11)	3/62 (5)

Abbreviations: ACR, American College of Rheumatology; BMI, body mass index (calculated as weight in kilograms divided by height in meters squared); EQ-5D-5L, EuroQol with 5 dimensions and 5 response levels; IA, intra-articular; ICOAP, intermittent and constant osteoarthritis pain; IM, intramuscular; IPAQ, International Physical Activity Questionnaire; KOOS, Knee Injury and Osteoarthritis Outcome Score; NRS, numeric rating scale; NSAID, nonsteroidal anti-inflammatory drug; OA, osteoarthritis; WOMAC, Western Ontario and McMaster Universities Osteoarthritis Index.

^a^ Score range, 0 to 100; 0 indicates extreme symptoms.

^b^ Score range, 0 to 100; 0 indicates no symptoms.

^c^ Score range, 0 to 10; 0 indicates no pain.

^d^ Score range, 0 to 10; 0 indicates no stiffness.

^e^ Score range, 0 to 10; 0 indicates no swelling.

^f^ Score range, 0 to 100; 0 indicates no pain.

^g^ Score range, −0.446 to 1.000; −0.446 indicates worst health-related quality of life.

^h^ Data on 1 IA participant missing.

^i^ Assessed on the injection day by the treating general practitioner.

^j^ Radiographs (taken within 1 year from baseline) were available for 131 patients (90%).

### Primary Outcome Measure

**Figure 2.**
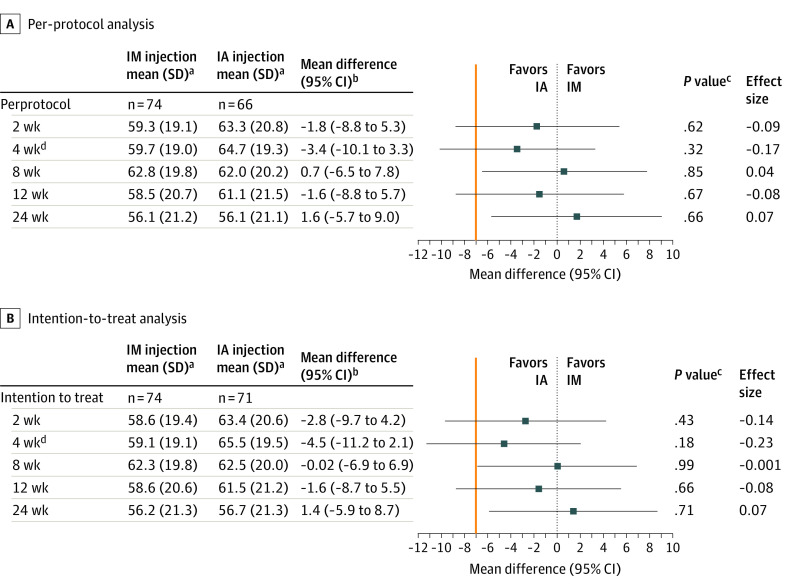
Knee Injury and Osteoarthritis Outcome Score Pain Score for Per-Protocol and Intention-to-Treat Analyses at All Follow-up Points Mean differences were calculated by treating the IA group as the reference. There were no missing values in the model covariates, and all patients were included in modeling. The noninferiority margin was −7 (orange vertical line). IA indicates intra-articular; IM, intramuscular. ^a^Unadjusted values. ^b^IA injection as reference; adjusted for baseline Knee Injury and Osteoarthritis Outcome Score, sex, presence of depression, and duration of knee osteoarthritis symptoms. ^c^*P* values for superiority tests. ^d^Primary time point.

In both groups, the KOOS pain score improved over the entire 24-week follow-up period; the greatest improvements were observed 8 weeks after the IM injection and 4 weeks after the IA injection. The mean improvements exceeded the minimal clinically important difference from 2 to 12 weeks within each group (Figure 2; eFigure in Supplement 2). At the primary time point (4 weeks), the estimated mean difference in the KOOS pain score between the 2 groups was −3.4 (95% CI, −10.1 to 3.3; effect size = −0.17). Noninferiority could not be declared because the lower limit of the 95% CI exceeded the noninferiority margin. The IM injection was found to be noninferior to the IA injection at 8 (mean difference, 0.7; 95% CI, −6.5 to 7.8) and 24 (mean difference, 1.6; 95% CI, −5.7 to 9.0) weeks, but not at 2 and 12 weeks. These results were robust to the sensitivity analysis in the intention-to-treat population (Figure 2).

### Secondary Outcomes

Both injections improved joint symptoms, function, stiffness, patient sport level, and quality of life over the entire 24-week follow-up period. The IM injection presented its greatest effectiveness at 8 weeks after injection, whereas the IA injection was most effective at 4 weeks in almost all secondary outcomes. There was no significant difference between the 2 groups at all time points for all the secondary outcomes (Table 2 and Table 3). These results were similar for the intention-to-treat population (eTable 1 and eTable 2 in Supplement 2).

**Table 2.** Results of the Linear Mixed Models With Repeated Measurements for Between-Group Differences Regarding Secondary Outcomes Based on Per-Protocol Analysis

Variable	Mean (SD)^a^		Mean difference (95% CI)^b^	*P* value^c^	Effect size
	IM (n = 72)	IA (n = 66)			
**KOOS** ^d^					
Symptoms					
2 wk	64.3 (15.4)	69.0 (17.2)	−3.0 (−8.7 to 2.8)	.31	−0.18
4 wk	65.9 (17.4)	70.1 (17.8)	−2.6 (−8.8 to 3.6)	.40	−0.15
8 wk	68.0 (17.9)	66.0 (18.0)	1.4 (−5.2 to 7.9)	.68	0.08
12 wk	64.3 (18.9)	65.0 (19.7)	−0.2 (−6.9 to 6.5)	.95	−0.01
24 wk	63.4 (20.3)	62.7 (20.0)	1.7 (−5.3 to 8.7)	.63	0.08
Function					
2 wk	63.3 (20.5)	67.6 (21.9)	−1.7 (−9.0 to 5.6)	.65	−0.08
4 wk	64.2 (20.9)	67.7 (20.1)	−1.6 (−8.8 to 5.6)	.66	−0.08
8 wk	67.8 (21.2)	64.7 (22.1)	3.1 (−4.5 to 10.8)	.42	0.14
12 wk	63.3 (22.6)	64.3 (21.1)	−0.4 (−7.9 to 7.1)	.92	−0.02
24 wk	60.6 (22.7)	60.0 (22.7)	3.0 (−4.8 to 10.9)	.45	0.13
Sport and recreation					
2 wk	26.3 (22.5)	27.9 (26.5)	0.4 (−8.2 to 9.0)	.92	0.02
4 wk	26.1 (22.5)	30.4 (26.3)	−3.0 (−11.4 to 5.5)	.49	−0.12
8 wk	30.7 (23.3)	27.2 (24.2)	4.4 (−3.9 to 12.7)	.30	0.18
12 wk	29.0 (24.7)	25.3 (25.4)	4.2 (−4.5 to 12.8)	.35	0.16
24 wk	23.5 (22.5)	24.2 (27.3)	0.9 (−7.9 to 9.6)	.84	0.04
Quality of life					
2 wk	39.6 (17.5)	42.7 (20.4)	0.4 (−6.1 to 7.0)	.89	0.02
4 wk	40.1 (19.5)	44.9 (20.4)	−1.4 (−8.2 to 5.5)	.69	−0.07
8 wk	44.5 (19.8)	45.1 (21.6)	1.4 (−5.7 to 8.5)	.70	0.07
12 wk	41.8 (20.7)	45.3 (21.5)	−1.1 (−8.2 to 6.0)	.76	−0.05
24 wk	39.3 (20.5)	41.0 (19.7)	0.9 (−5.9 to 7.7)	.79	0.04
**WOMAC** ^e^					
Pain					
2 wk	35.3 (20.8)	30.2 (21.7)	3.1 (−4.4 to 10.6)	.41	0.14
4 wk	33.9 (20.8)	29.8 (20.1)	2.9 (−4.3 to 10.1)	.43	0.14
8 wk	30.7 (21.0)	32.5 (20.4)	−1.4 (−8.8 to 6.0)	.70	−0.07
12 wk	35.2 (21.7)	33.0 (22.5)	1.6 (−6.1 to 9.2)	.69	0.07
24 wk	38.0 (22.4)	37.8 (22.3)	−1.1 (−8.9 to 6.6)	.77	−0.05
Function					
2 wk	36.7 (20.5)	32.4 (21.8)	1.7 (−5.6 to 9.0)	.65	0.08
4 wk	35.8 (20.9)	32.3 (20.1)	1.6 (−5.6 to 8.8)	.66	0.08
8 wk	32.2 (21.2)	35.3 (22.1)	−3.1 (−10.8 to 4.5)	.42	−0.14
12 wk	37.7 (22.6)	35.7 (21.1)	0.4 (−7.1 to 7.9)	.92	0.02
24 wk	39.3 (22.7)	40.0 (22.7)	−3.0 (−10.9 to 4.8)	.45	−0.13
Stiffness					
2 wk	44.7 (18.7)	41.0 (22.5)	2.3 (−5.0 to 9.6)	.53	0.11
4 wk	41.8 (21.7)	38.2 (23.5)	2.4 (−5.5 to 10.3)	.55	0.10
8 wk	39.6 (23.5)	43.4 (23.6)	−3.1 (−11.5 to 5.3)	.47	−0.13
12 wk	44.0 (23.5)	44.8 (23.9)	−0.9 (−9.2 to 7.3)	.83	−0.04
24 wk	45.3 (24.3)	49.2 (23.9)	−5.0 (−13.3 to 3.3)	.24	−0.21
Total					
2 wk	37.1 (19.8)	32.6 (21.1)	2.0 (−5.1 to 9.1)	.57	0.10
4 wk	35.9 (20.2)	32.3 (19.4)	2.0 (−5.0 to 9.0)	.57	0.10
8 wk	32.5 (20.5)	35.4 (21.1)	−2.7 (−10.1 to 4.7)	.47	−0.13
12 wk	37.7 (21.7)	35.9 (20.7)	0.6 (−6.7 to 7.8)	.87	0.03
24 wk	39.6 (21.9)	40.3 (21.9)	−2.8 (−10.4 to 4.8)	.47	−0.13
**NRS pain** ^f^					
2 wk	4.8 (2.2)	4.4 (2.6)	0.3 (−0.5 to 1.1)	.45	0.13
4 wk	5.0 (2.3)	4.3 (2.3)	0.6 (−0.2 to 1.4)	.11	0.26
8 wk	4.5 (2.3)	5.1 (2.4)	−0.6 (−1.4 to 0.3)	.12	−0.26
12 wk	5.2 (2.2)	5.0 (2.5)	0.3 (−0.5 to 1.1)	.52	0.13
24 wk	5.6 (2.2)	5.6 (2.3)	−0.02 (−0.8 to 0.8)	.96	−0.01
**ICOAP** ^g^					
Constant pain					
2 wk	29.6 (21.8)	23.3 (22.7)	4.5 (−3.3 to 12.3)	.26	0.20
4 wk	31.2 (23.1)	25.8 (21.5)	4.3 (−3.6 to 12.1)	.29	0.19
8 wk	28.0 (23.3)	29.1 (22.5)	−0.5 (−8.8 to 7.9)	.91	−0.02
12 wk	33.1 (22.1)	29.2 (23.0)	4.3 (−3.7 to 12.3)	.29	0.19
24 wk	33.1 (22.6)	32.5 (23.1)	0.1 (−8.1 to 8.3)	.98	0.004
Intermittent pain					
2 wk	34.3 (21.3)	26.3 (21.9)	5.2 (−2.3 to 12.8)	.17	0.24
4 wk	36.2 (20.7)	30.3 (21.7)	4.1 (−3.4 to 11.5)	.28	0.19
8 wk	33.5 (21.9)	32.8 (20.5)	0.3 (−7.3 to 7.9)	.93	0.01
12 wk	35.8 (21.7)	32.6 (24.1)	2.6 (−5.4 to 10.7)	.52	0.11
24 wk	38.4 (20.3)	36.2 (22.8)	1.0 (−6.6 to 8.7)	.79	0.05
Total score					
2 wk	32.1 (20.8)	25.0 (21.6)	5.0 (−2.4 to 12.4)	.19	0.23
4 wk	34.0 (21.0)	28.2 (21.1)	4.2 (−3.2 to 11.6)	.26	0.20
8 wk	31.0 (21.9)	31.1 (20.9)	0.04 (−7.7 to 7.8)	.99	0.002
12 wk	34.6 (20.9)	31.0 (23.3)	3.5 (−4.3 to 11.3)	.37	0.16
24 wk	36.0 (20.3)	34.5 (22.3)	0.7 (−7.0 to 8.3)	.87	0.03
**EQ-5D-5L** ^h^					
4 wk	0.67 (0.26)	0.74 (0.21)	−0.03 (−0.12 to 0.05)	.42	−0.12
24 wk	0.67 (0.27)	0.68 (0.25)	0.02 (−0.07 to 0.11)	.65	0.08

Abbreviations: EQ-5D-5L, EuroQol with 5 dimensions and 5 response levels; IA, intra-articular; ICOAP, intermittent and constant osteoarthritis pain; IM, intramuscular; KOOS, Knee Injury and Osteoarthritis Outcome Score; NRS, numeric rating scale; WOMAC, Western Ontario and McMaster Universities Osteoarthritis Index.

^a^ Observed means and SDs, unadjusted values. There were no missing values in the model covariates, and all patients (138) were included in modeling.

^b^ Intra-articular injection as reference, adjusted for baseline score, sex, presence of depression, and duration of knee osteoarthritis symptoms. Calculated based on estimated marginal means.

^c^ *P* values for superiority tests.

^d^ Score range, 0 to 100; 0 indicates extreme symptoms.

^e^ Score range, 0 to 100; 0 indicates no symptoms.

^f^ Score range, 0 to 10; 0 indicates no pain.

^g^ Score range, 0 to 100; 0 indicates no pain.

^h^ Score range, −0.446 to 1.000; −0.446 indicates worst health-related quality of life.

**Table 3.** Results of Generalized Estimating Equations With Repeated Measurements for Between-Group Differences Regarding Responders and Perceived Recovery Based on a Per-Protocol Analysis

Characteristic	No./total No. (%)^a^		OR (95% CI)^b^	*P* value^c^
	IM injection	IA injection		
**OMERACT-OARSI responder criteria**				
2 wk	34/68 (50)	41/65 (63)	0.8 (0.4-1.8)	.61
4 wk	33/69 (48)	45/66 (68)	0.6 (0.3-1.2)	.12
8 wk	36/65 (55)	37/66 (56)	1.3 (0.6-2.7)	.45
12 wk	27/67 (40)	36/65 (55)	0.7 (0.4-1.6)	.43
24 wk	19/67 (28)	32/65 (49)	0.5 (0.2-1.1)	.10
**Perceived recovery**				
2 wk	41/68 (60)	50/65 (77)	0.6 (0.3-1.3)	.18
4 wk	43/69 (62)	50/66 (76)	0.6 (0.3-1.3)	.22
8 wk	40/65 (62)	41/66 (62)	1.0 (0.5-2.2)	.90
12 wk	31/67 (46)	37/65 (57)	0.7 (0.3-1.5)	.35
24 wk	27/67 (40)	29/65 (45)	0.9 (0.5-1.9)	.82

Abbreviations: IA, intra-articular; IM, intramuscular; OR, odds ratio.

^a^ Observed and unadjusted values. There were no missing values in the model covariates, and all patients (138) were included in modeling.

^b^ Intra-articular injection as reference; adjusted for baseline Knee Injury and Osteoarthritis Outcome Score pain score, sex, presence of depression, and duration of knee osteoarthritis symptoms. Calculated based on estimated marginal percentages.

^c^ *P* values for superiority tests.

### Adverse Events and Cointerventions

At 2 weeks’ follow-up, 24 patients (33%) in the IM group reported 27 adverse events and 28 patients (42%) in the IA group reported 38 adverse events. The most frequently reported adverse events were hot flush (IM, 7 [10%] vs IA, 14 [21%]) and headache (IM, 10 [14%] vs IA, 12 [18%]), and all events were classified as nonserious (eTable 3 in Supplement 2). The results of cointerventions are presented in eTable 4 in Supplement 2. Patients in both groups reported less use of oral analgesics after the injections (eg, nonsteroidal anti-inflammatory drugs at 4 weeks: IM, 9 [13%] vs IA, 7 [11%]); 4 patients (6%) in the IA group received an additional IA glucocorticoid injection within 8 weeks vs none in the IM group. Within 24 weeks, 4 patients (6%) in the IM group and 9 patients (14%) in the IA group had received an additional IA glucocorticoid injection.

### Subgroup Analysis

The baseline pain severity had no significant interactive effect on KOOS pain scores between the 2 groups (estimate, −2.5; 95% CI, −12.4 to 7.4; *P* = .62). The results were consistent in the intention-to-treat population.

## Discussion

Despite clinically relevant improvements in both groups, the trial findings did not demonstrate the noninferiority of IM glucocorticoid injection in reducing OA knee pain at 4 weeks. This result could be partially explained by the finding that IM injection presented a peak effect at 8 weeks vs 4 weeks for IA injection. Accordingly, the IM injection reached the noninferiority level at 8 and 24 weeks after administration, while the effects at 24 weeks were small for both injections. Moreover, patients with IA injection reported slightly more adverse events, although none were serious.

To our knowledge, this is the first randomized clinical trial evaluating the effectiveness of an IM glucocorticoid injection for knee OA. The results are consistent with a previous trial of IM injection in patients with hip OA, with IM injection showing clinically relevant effects compared with placebo from 2 to 12 weeks and had the greatest effect approximately 6 weeks after injection.^16^ Based on these results, patients receiving an IM glucocorticoid injection are likely to experience a continuous reduction in knee pain within 8 weeks, in contrast with IA injection, which provides substantial short-term symptom relief (2-4 weeks). The mechanism behind this difference could be related to pharmacokinetics. Given that suppression of joint inflammation by IA injection of corticosteroids is associated with a lower level of knee pain,^27^ an IM injection may need longer to reach an adequate concentration of corticosteroids in the knee compared with an IA injection. However, this hypothesis needs to be tested in further studies.

The analgesic effect of the IA injection presented in this trial is similar to the previous trials of Conaghan et al^5^ and Deyle et al,^28^ but larger than the effect (at 2 weeks) reported in the trial of Henriksen et al.^22^ A possible reason for the difference could be that Henriksen et al^22^ recruited a group of patients with less pain at baseline than in this present trial, and patients with milder pain were reported to experience a smaller benefit from glucocorticoid injection.^29^

For clinical practice, interpretation should also include the adverse events, patient preference, and clinician’s skills. Combined with the results of a previous trial, a single IM injection of triamcinolone, 40 mg, should be considered safe because no injection-related serious adverse event was reported in patients with either hip or knee OA.^16^ In addition, according to the baseline assessment of this study, more patients preferred IM to IA injection (47% vs 19%). These findings should be mainly considered as slight preferences and might not be extrapolated to the general population, because patients with strong preferences would probably have declined to participate in this trial. Nevertheless, this selection reflects the fact that the IM injection is a preferable approach in some cases from the perspective of the patients. Furthermore, effect sizes of between-group differences were small at all time points, and no significant differences were found in any of the primary and secondary outcomes, including joint pain, function, stiffness, patient sport level, and quality of life. It may be best to inform patients on these outcomes, especially when clinicians feel incompetent in administering an IA injection. Taken together, the findings of this trial suggest that a shared decision-making process between clinicians and patients with knee OA would be useful when a glucocorticoid injection is indicated.

### Strengths and Limitations

This study has several strengths. First, we recruited the targeted number of patients from our sample-size calculation and had high adherence and follow-up rates. As a result, statistical inferences were consistent in the per-protocol and intention-to-treat populations. Second, we administered IM injections at the ventrogluteal region, which contains a thin layer of subcutaneous fat and helped decrease the possibility of subcutaneous injection, especially for patients who were overweight.^17,30^ Third, the multicenter design strengthened the generalizability of the results to other primary care clinics.

The trial has limitations. First, as a pragmatic trial, it was practically impossible to blind GPs and patients to the treatment allocation, and no placebo-controlled group was incorporated, so the measured effectiveness of the 2 injections might incorporate part of a placebo effect. However, as previously reported, the placebo effect of injection therapy for knee OA seems mainly derived from the use of the IA delivery methods.^31,32^ Therefore, IM injection is likely to have a smaller placebo effect than IA injection. This hypothesis is supported by the previous trial in which IM injection presented a minimal placebo effect in patients with hip OA.^16^ Moreover, pragmatic trials are designed for simulating clinical effectiveness; elimination of the placebo effect would underestimate the real benefit.^33^ Second, although the noninferiority margin of 7 points was prespecified, taking clinical relevance into account, it was a subjective choice. As described in the CONSORT statement and previous noninferiority trials, an evidence-based margin rarely exists.^18,34,35^ Third, this trial was designed to assess the effectiveness of a single injection; however, in total, 4 patients (6%) in the IM group and 9 patients (14%) in the IA group received an additional IA glucocorticoid injection before 24 weeks’ follow-up.

## Conclusions

The findings of this trial suggest that, among patients in primary care settings with symptomatic knee OA, an IM glucocorticoid injection could present an inferior effect in reducing pain at our primary end point of 4 weeks, compared with an IA injection. An IM injection is noninferior to IA injection at 8 and 24 weeks after injection, but not at 2 and 12 weeks. Both types of injection should be considered effective strategies, and this trial provides evidence for shared decision-making between clinicians and patients, taking into account the advantages and disadvantages of both treatment strategies.

## Notes

Supplement 1.
Trial Protocol

Supplement 2. **eTable 1.** Results of the Linear Mixed Models With Repeated Measurements for Between-Group Differences Regarding Secondary Outcomes Based on an Intention-to-Treat Analysis **eTable 2.** Results of Generalized Estimating Equations With Repeated Measurements for Intergroup Differences Regarding Responders and Perceived Recovery Based on an Intention-to-Treat Analysis **eTable 3.** Adverse Events at 2 Weeks’ Follow-up **eTable 4.** Cointerventions of Patients in the Two Groups **eFigure.** Unadjusted KOOS Pain Scores of Patients Included in Per-Protocol Analysis Over the Entire Follow-up

Supplement 3.
Data Sharing Statement
