# Supplementary material for: Effect of Intramuscular vs Intra-articular Glucocorticoid Injection on Pain Among Adults With Knee Osteoarthritis: The KIS Randomized Clinical Trial
Source: JAMA Netw Open. 2022 Apr 5;5(4):e224852. doi: 10.1001/jamanetworkopen.2022.4852 (PMC8984774; doi:10.1001/jamanetworkopen.2022.4852)
Supplement: Supplement 1. — Trial Protocol [file jamanetwopen-e224852-s001.pdf]

Effectiveness of intramuscular gluteal glucocorticoid injection versus intra-articular glucocorticoid injection in knee osteoarthritis: design of a multicenter randomized controlled non-inferiority trial

Trial registration

This trial is registered in the Dutch Trial Registry: number NTR6968.

Background

The knee is symptomatically the most frequent affected joint in osteoarthritis (OA). In the Netherlands and other Western countries this is mainly managed by general practitioners[1]. The prevalence of knee OA in general practice was estimated around 40.2 per 1000 patient years (28.9 men; 51.4 women) in 2018[2].

For OA patients, pain and disability are the most important reasons to seek care of a health professional[3-5]. If patients are not responding satisfactorily to paracetamol, NSAIDs and non-drug treatment, or in cases of interim aggravation, the evidence-based guideline from the Dutch College of General Practitioners on 'Non-traumatic knee complaints' suggests intra-articular (IA) glucocorticoid injection with 20 to 40 mg triamcinolone acetonide[6]. In several international guidelines IA glucocorticoid injection is also recommended for these abovementioned indications[7, 8].

Despite the long-standing frequent application of IA glucocorticoids, there is an ongoing debate about their effectiveness and safety[14, 15]. IA glucocorticoid injection in patients with knee OA leads to a moderate improvement in pain, but only in the short term (1 to 6 weeks after injection)[14].. IA injection has a small risk of the serious adverse reaction of septic arthritis on the short term[16]. In recent literature there is controversy over the chondrotoxicity of IA glucocorticoids on the longer term[17-19].

An additional obstacle in the way of IA injection is that GPs might feel incompetent to administer this type of injection due to lack of training and experience[22]. Due to the GP's restraint, knee OA patients who could benefit from IA injection might not always receive timely injection[22].

Intramuscular (IM) glucocorticoid injection could be a valuable alternative treatment for IA glucocorticoid injection for patients with knee OA. IM administration eliminates the risk of septic arthritis and direct cartilage toxicity. The favorable effect of IM glucocorticoids on musculoskeletal pain has been studied originally in patients with rotator cuff disease and is used for rheumatoid arthritis[23, 24]. In a recent study from our study group, a clinical relevant and statistical significant difference in pain reduction was found for IM glucocorticoid injection compared to placebo in patients with hip OA[26]. Remarkably, the effect of the IM injection lasted at least 12 weeks. As of now no direct comparison between the effectiveness of IM and IA glucocorticoid injection in OA has been made.

We will perform a randomized controlled two-parallel-groups trial in patients with knee OA included from general practices, assessing the non-inferiority of an IM gluteal glucocorticoid injection compared to an IA glucocorticoid knee injection at four weeks follow-up. We hypothesize that IM gluteal glucocorticoid injection is non-inferior to IA glucocorticoid injection in reducing knee pain 4 weeks after injection, with potentially a longer lasting effect for at least 12 weeks.

Primary objective

The primary objective is to assess whether IM gluteal glucocorticoid injection is non-inferior to IA knee glucocorticoid injection in reducing knee pain, measured with the Knee injury and Osteoarthritis Outcome Score (KOOS) pain subscale, in patients with knee OA in general practice at 4 weeks after injection.

Secondary objectives

The study will evaluate the differences in reported adverse events frequency and co-interventions of patients allocated to an IM gluteal glucocorticoid injection or to an IA glucocorticoid injection. Differences between the two treatment groups in several outcome measures related to knee recovery, on short and longtime follow-up (2-24 weeks) and quality of life will be measured (see table 2).

Methods

Design

This study is a pragmatic randomized controlled trial with two parallel groups with a follow-up of 24 weeks (see figure 1). The Medical Ethics Committee of Erasmus MC University Medical Center Rotterdam approved this trial (MEC 2017-563). All patients will give written informed consent prior to data collection.

Patient selection

Patients with OA of the knee will be recruited by participating GPs located in the south-west of the Netherlands. Patient selection can take place in two different ways. The electronic patient files will be scanned for the International Classification of Primary Care (ICPC) codes L90 (knee OA) and L15 (knee symptoms/complaints) in order to identify eligible patients who have no contraindications for participation in this study. See table 1 for the inclusion- and exclusion

criteria. Patients who are identified with the ICPC code L15 are only selected if there is a note by the GP or a radiology report that mentions ‘osteoarthritis’ or ‘cartilage degeneration’. The second way is that GPs are asked to invite patients who consult them for knee OA to participate in the study. The GPs are asked to screen the inclusion- and exclusion criteria for all patients and will also directly assess whether there is an indication for glucocorticoid injection. An intra-articular glucocorticoid injection is recommended in guidelines for patients with knee osteoarthritis as an option for a flare of knee pain and/or for those who are not responding well to paracetamol or Non-Steroidal Anti-Inflammatory Drug (NSAID) treatment. Patients who have had an IA injection in the knee during the previous 6 months will be excluded, since a /prolonged treatment effect of 24 weeks after injection has been described[27]. Patients with diabetes mellitus on insulin therapy or with a poor glycemic control (as assessed by their GP) cannot participate, as they might be at risk of prolonged blood glucose level elevation after glucocorticoid injection [28, 29]. Patients who have been referred to an orthopedic surgeon will also be excluded from participation considering that these patients could become candidates for total knee or hip arthroplasty during the follow-up period of the study. The risk of periprosthetic joint infection (PJI) is increased in patients who received an IA glucocorticoid injection in the 3 months prior to arthroplasty[30, 31]. It is not known if IM glucocorticoid injection increases the risk of PJI.

For patients who are selected via their electronic patient file, the actual amount of knee pain they experience is not known by their GP. These patients’ knee pain level will be checked over the phone by the researchers (given that the patient is willing to participate in the study, see figure 1). A minimum score of 3 on the numerical rating scale (NRS, 0-10; 0=no knee pain) is required in order to participate in the trial. If a patient has an NRS knee pain score <3, the patient will be asked to contact the research team in case of future increase in knee pain. In case a patient has bilateral knee OA, the most painful knee is selected as the ‘study knee’.

Procedures

The GPs will inform all eligible patients about the study in writing. Patients will receive information about the study and a reply card. Once the research team receives a reply card with a positive response from a patient, a researcher will contact this patient by telephone. The researcher will ask the patient about the severity of knee pain averaged over the past week. To all patients with a pain score ≥3 additional written information about the study will be sent. Several days later, the researcher will contact the patients again to further explain the study and to answer remaining questions. Patients who are interested to participate will be asked to give written informed consent. After the patients have given this consent, the baseline questionnaire are sent to these patients.

After completion of the baseline questionnaire, the patient will be randomly allocated to one of the two treatment groups. The GP and the patient will be informed about the outcome of randomization. The GP will prepare and administer the allocated injection. We aim to have the injection administered within one week after completion of the baseline questionnaire. This is to ascertain the pain score at the moment of injection is unchanged or close to the baseline score. Change in pain since baseline can never lead to exclusion of a trial participant.

The GP will complete a case report form at the patients' visit for administration of the injection. This report form asks for the American College of Rheumatology criteria for clinical diagnosis of knee OA, location of injection, severity of knee pain averaged over the past week and the batch number of the triamcinolone acetonide [32].

All patients will be referred for an AP weight-bearing X-ray of the studied knee if an X-ray has not been made in the 12 months prior to enrolment. The 12 month period was chosen as the risk of annual radiographic OA progression by at least one Kellgren-Lawrence (K-L) grade has been estimated low[33]. Therefore, the X-ray does not have to be obtained directly at baseline, but

will be made during follow-up. We consider the radiograph necessary in order to facilitate comparison between our study population and patient data collected in previous studies. Two researchers will independently assess the X-rays to grade radiographic knee OA, using the K-L classification[34].

Randomization

After the patients sign informed consent, they will be randomized and receive their allocated intervention. After informed consent the patient will be assigned an unique trial number.

An independent researcher, who will not meet or contact the patients, has prepared a computer generated randomization list using 1:1 allocation and random blocks of 8, 6 or 4 in order to ensure concealment of allocation.

Blinding

Due to the pragmatic nature of this trial, the patient and the GP are not blinded for treatment allocation. The researcher involved in data analysis will be blinded for treatment allocation.

Intervention

The investigational treatment will consist of 40 mg triamcinolone acetonide (Kenacort-A 40). The chosen dosage of glucocorticoid is based on clinical experience[35]. No local anesthetic will be added to the injection. The GP will inject either IA in the knee joint or IM in the ipsilateral ventrogluteal region. All participating GPs will be invited for an optional IA knee injection training under supervision of an experienced orthopedic surgeon (PKB).

The superolateral IA injection approach to the knee will be used (just below the upper border of the patella and 1cm lateral to the lateral border of the patella, see figure 2B). This approach has

an accuracy of 91% for needle placement in the IA space of the knee and is recommended by the Dutch College of GPs[6, 36].

The IM injection will be administered in the ventrogluteal region (the region between the iliac crest, greater trochanter of the femur and anterior iliac spine, see figure 2A) in order to prevent injury to the sciatic nerve[37, 38]. Moreover, administration in the ventrogluteal region diminishes the possibility of subcutaneous injection in overweight patients since the layer of subcutaneous fat is less thick in the ventrogluteal region compared to the dorsogluteal region[39]. It is well known that knee OA is more common in patients with a BMI >27kg/m^2 [40].

Co-interventions are allowed during the follow-up and will be monitored.

Measurements

Baseline measurements

See table 2 for an overview of baseline measurements. Demographic measurements consist of age, sex, educational level, and daily occupation. Also self-reported length and weight are reported. Medication use for knee OA ..

Intermittent and constant OA pain will be measured with the Intermittent and Constant OsteoArthritis Pain score (ICOAP:0-100; 0=no pain)[43]. Knee complaint characteristics (duration of symptoms at baseline, sensation of swelling in the knee as an indicator of flare-up) will be recorded. Knee pain severity averaged over the last week will be measured with an 11-point numerical rating scale (NRS:0-10; 0=no pain). Health related Quality of Life (QoL) will be measured with the EQ-5D-5L (scores ranging from -0.446=worst health related QoL to 1.0=perfect health related QoL)[44, 45] Co-morbidity will be measured at baseline using a multiple choice format where patients can select multiple answers and add a missing comorbid disease.

Also measured at baseline will be physical activity over the past week (IPAQ short), neuropathic pain (modified painDETECT questionnaire), patients' preferred injection site (knee or ventrogluteal area) and patients' expected treatment response[41, 42] Patients' preferred injection site has no influence on treatment allocation.

Follow-up measurements

Outcomes are measured at 2, 4, 8, 12 and 24 weeks after administration of the injection using digital questionnaires. Patients without an electronic mailbox will receive paper questionnaires. The primary outcome is patient reported severity of pain at 4 weeks after injection measured with the KOOS pain subscale (0-100; 0=extreme pain). Secondary study endpoints are listed in table 2. Disability will be measured with the KOOS Function in daily living (0-100; 0=extreme problems)[46].. Patients' perceived recovery is measured with a 7-point Likert scale that will be dichotomized in recovered ('complete recovery', 'much improved', 'slightly improved') and not-recovered ('no change', 'slightly worse', 'much worse',' 'worse than ever')[26]. Percentage responders is defined by the OMERACT-OARSI criteria: High improvement (≥50%) in KOOS pain subscale or in KOOS function in daily living subscale and absolute increase ≥20 points in KOOS pain subscale or function in daily living subscale, if not then improvement in at least 2 of the 3 following domains: 1) ≥20% improvement in KOOS pain subscale and ≥10 points increase in KOOS pain subscale, 2) ≥20% improvement in KOOS function in daily living subscale and ≥10 points increase in KOOS function in daily living subscale, 3) ≥20% increase in global score and ≥10 points increase in global score. In this study patients' global score will be measured with a patients' perceived recovery score measured on a 7-point Likert scale. This domain is considered improved if a patient fills in 'complete recovery', 'much improved', or 'slightly improved'[47]. Two weeks after administration of the injection patients are asked to report adverse

events. Also, follow-up questionnaires at all time points ask about hospitalization in order to monitor Serious Adverse Events. . Co-interventions including medication, non-drug therapies such as physiotherapy, referrals and surgery will be measured with the modified medical consumption questionnaire of the Institute for Medical Technology Assessment (iMCQ)[48]. Experienced painfulness of injection will be registered two weeks after glucocorticoid injection.

Sample size

For the sample size calculation, we used data from the study of Henriksen et al. that evaluated the clinical benefits of an IA glucocorticoid injection given before exercise therapy in patients with knee OA[49]. The results of the study reported a baseline standard deviation of 16 for the KOOS pain (recommended by the KOOS for sample size calculations is a standard deviation of 15). The minimal important difference (=non-inferiority margin) between both treatment groups of the patient reported outcome KOOS (0-100) was set at 7 points (effect size of 0.44)[50, 51].

For the non-inferiority of an IM gluteal glucocorticoid injection compared to an IA knee glucocorticoid injection, we will need 65 patients per group, using a power of 80%, an alpha of 5%, a non-inferior margin of 7 and a SD of 16. Taking into account a loss to follow-up of 5%, this trial needs to include (2*65)+(0.05*2*65)=~140 patients. We expect a low percentage of loss to follow-up because of the relative short follow-up period of 24 weeks and our prior experience in glucocorticoid trials[25, 26].

Statistical analysis plan

Imbalance in the baseline variables of the two treatment arms might occur after randomization. This is problematic if the imbalanced variable is related to the outcome variable, as this could lead to confounding[52, 53]. In case imbalance occurs we will adjust for relevant variables. Descriptive statistics will be used to describe patients' and complaints characteristics at baseline. Analyses will be adjusted for variables that hamper the baseline interchangeability of groups when there are clinical relevant differences between groups of over 10%.

The primary outcome is patient reported severity of pain at 4 weeks after injection measured with the KOOS pain (0-100; 0=extreme pain). We will use a non-inferiority design to assess if an IM gluteal injection is non-inferior to an IA knee injection with regard to this outcome. In non-inferiority comparisons intention-to-treat analysis can bias towards the null and could increase

type I error; the risk of falsely claiming non-inferiority[54]. Therefore, in addition a per-protocol analysis will be performed as sensitivity analysis to assess consistent non-inferiority. Included in the per-protocol analysis will be patients who received the assigned injection and reported the KOOS pain at 4 weeks follow-up. In case a patient from the IM injection group receives an additional IA injection earlier than 6 weeks after the study injection, this will be considered as a protocol violation and the patient will be excluded from the per-protocol analysis. The reason for this is that the guideline from the Dutch College of General Practitioners recommends to leave at least six weeks between two consecutive injections[6]. Non-inferiority of the IM injection will also be assessed using both intention-to-treat and per-protocol analysis at 2, 8, 12 and 24 weeks follow-up and for the outcome KOOS pain. For the other outcome measures we will calculate mean differences.

We expect 10-15% of missing data (incompletely filled in paper questionnaires and loss to follow-up). We will contact the patients to pose them the missing questions again in order to minimize missing data for the primary outcome. Multiple imputations will be performed for missing values (incompletely filled in questionnaires), creating at least five imputed datasets.

Linear mixed models with repeated measures will be used for the primary outcome as this is a continuous variable. To model the covariance of repeated measures by patients, a structure will be chosen with the lowest Akaike's information criterion. Fixed effects will be time, and time by treatment. Analysis will be adjusted for relevant confounders. Relevant confounder is defined as prognostic variable and has clinically relevant differences between groups of over 10%. Prognostic variables are determined by prior knowledge, and include baseline value of the outcome, baseline BMI, sex, diabetes, duration of the symptoms and depression for this study.

Linear mixed models with repeated measures will be used for the continuous secondary outcomes: KOOS, NRS, WOMAC, ICOAP, and EQ-5D-5L. Generalized estimating equations

analyses with repeated measures will be performed for the dichotomous outcomes: patients' perceived recovery (7 point Likert scale), and the OMERACT-OARSI responder criteria. The analyses will be adjusted for baseline KOOS pain score and the same baseline covariates as in the linear mixed models. Before generalized estimating equations analyses, multiple imputations will be performed for missing values of secondary study parameters, creating at least five imputed datasets.

When patients underwent a total knee replacement surgery, data of these patients will be included up to the date of surgery. Missing data for secondary outcome measurements will be handled similarly as missing data for the primary outcome.

Subgroup analysis

An explorative, pre-defined, subgroup analysis will be performed assessing the interaction effects between injections regarding the severity of knee pain at baseline (NRS pain score of >=7 versus <7) on the primary outcome[56]. Generalized estimating equations will be used to analyze differences between groups concerning adverse events, medical consumption and medication usage. Change in medication usage will be defined as yes or no increase in pain medication and yes or no decrease in pain medication use.

<u>Subsequent amendments</u>

Patients were only followed from 8 weeks for reporting additional injections, we did not have the data of additional injections within 6 weeks. Therefore, we modified it into additional injections within 8 weeks while doing the analysis.

The proportion of missing value was small, therefore, no imputation was done.

An 'unstructured' covariance structure was chosen as it resulted in the lowest Akaike's information criterium. See **Table3** for AIC of the tested covariance type.

Competing interests

The authors declare that they have no conflict of interest.

Acknowledgements

Financial support was received from ZonMw (The Netherlands Organisation for Health Research and Development).

References

1. Oliveria, SA, Felson, DT, Reed, JI, Cirillo, PA, Walker, AM: Incidence of symptomatic hand, hip, and knee osteoarthritis among patients in a health maintenance organization. *Arthritis Rheum,* 38**:** 1134-1141, 1995.
2. NIVEL: Incidence and prevalence knee osteoarthritis in general practice 2018. https://wwwnivelnl/nl/nivel-zorgregistraties-eerste-lijn/incidenties-en-prevalenties, Assessed September 2019.
3. Paskins, Z, Sanders, T, Hassell, AB: What influences patients with osteoarthritis to consult their GP about their symptoms? A narrative review. *BMC Fam Pract,* 14**:** 195, 2013.
4. Rosemann, T, Wensing, M, Joest, K, Backenstrass, M, Mahler, C, Szecsenyi, J: Problems and needs for improving primary care of osteoarthritis patients: the views of patients, general practitioners and practice nurses. *BMC Musculoskelet Disord,* 7**:** 48, 2006.
5. Felson, DT, Neogi, T: Challenges for Osteoarthritis Trials. *Arthritis Rheumatol*, 2018.
6. Dutch Association of General Practitioners: NHG-guideline Non-traumatic knee complaints (In Dutch). 2016.
7. National Institute for Health and Care Excellence (NICE): Osteoarthritis: care and management. https://wwwniceorguk/guidance/cg177 Assessed October 2016.
8. McAlindon, TE, Bannuru, RR, Sullivan, MC, Arden, NK, Berenbaum, F, Bierma-Zeinstra, SM, Hawker, GA, Henrotin, Y, Hunter, DJ, Kawaguchi, H, Kwoh, K, Lohmander, S, Rannou, F, Roos, EM, Underwood, M: OARSI guidelines for the non-surgical management of knee osteoarthritis. *Osteoarthritis Cartilage,* 22**:** 363-388, 2014.
9. Berenbaum, F: Osteoarthritis as an inflammatory disease (osteoarthritis is not osteoarthrosis!). *Osteoarthritis Cartilage,* 21**:** 16-21, 2013.
10. de Lange-Brokaar, BJ, Ioan-Facsinay, A, Yusuf, E, Kroon, HM, Zuurmond, AM, Stojanovic-Susulic, V, Nelissen, RG, Bloem, JL, Kloppenburg, M: Evolution of synovitis in osteoarthritic knees and its association with clinical features. *Osteoarthritis Cartilage,* 24**:** 1867-1874, 2016.
11. Conaghan, PG, D'Agostino, MA, Le Bars, M, Baron, G, Schmidely, N, Wakefield, R, Ravaud, P, Grassi, W, Martin-Mola, E, So, A, Backhaus, M, Malaise, M, Emery, P, Dougados, M: Clinical and ultrasonographic predictors of joint replacement for knee osteoarthritis: results from a large, 3-year, prospective EULAR study. *Ann Rheum Dis,* 69**:** 644-647, 2010.
12. Collins, JE, Losina, E, Nevitt, MC, Roemer, FW, Guermazi, A, Lynch, JA, Katz, JN, Kent Kwoh, C, Kraus, VB, Hunter, DJ: Semiquantitative Imaging Biomarkers of Knee Osteoarthritis Progression: Data From the Foundation for the National Institutes of Health Osteoarthritis Biomarkers Consortium. *Arthritis Rheumatol,* 68**:** 2422-2431, 2016.
13. Felson, DT, Niu, J, Neogi, T, Goggins, J, Nevitt, MC, Roemer, F, Torner, J, Lewis, CE, Guermazi, A, Group, MI: Synovitis and the risk of knee osteoarthritis: the MOST Study. *Osteoarthritis Cartilage,* 24**:** 458-464, 2016.
14. Juni, P, Hari, R, Rutjes, AW, Fischer, R, Silletta, MG, Reichenbach, S, da Costa, BR: Intra-articular corticosteroid for knee osteoarthritis. *Cochrane Database Syst Rev***:** CD005328, 2015.
15. Liu, SH, Dube, CE, Eaton, CB, Driban, JB, McAlindon, TE, Lapane, KL: Longterm Effectiveness of Intraarticular Injections on Patient-reported Symptoms in Knee Osteoarthritis. *J Rheumatol,* 45**:** 1316-1324, 2018.
16. Charalambous, CP, Tryfonidis, M, Sadiq, S, Hirst, P, Paul, A: Septic arthritis following intra-articular steroid injection of the knee--a survey of current practice regarding antiseptic technique used during intra-articular steroid injection of the knee. *Clin Rheumatol,* 22**:** 386-390, 2003.

17. McAlindon, TE, LaValley, MP, Harvey, WF, Price, LL, Driban, JB, Zhang, M, Ward, RJ: Effect of Intra-articular Triamcinolone vs Saline on Knee Cartilage Volume and Pain in Patients With Knee Osteoarthritis: A Randomized Clinical Trial. *JAMA,* 317**:** 1967-1975, 2017.
18. Zeng, C, Lane, NE, Hunter, DJ, Wei, J, Choi, HK, McAlindon, TE, Li, H, Lu, N, Lei, G, Zhang, Y: Intra-articular corticosteroids and the risk of knee osteoarthritis progression: results from the Osteoarthritis Initiative. *Osteoarthritis Cartilage,* 27**:** 855-862, 2019.
19. Conaghan, PG: Corticosteroids and osteoarthritis progression: a confounded issue. *Osteoarthritis Cartilage,* 27**:** e5-e6, 2019.
20. Brandt, K: Management of osteoarthritis. In: *Kelley's Textbook of Rheumatology 6th Edition.* edited by RUDDY, S., HARRIS JR, E. D., SLEDGE, C. B., Philadelphia, W.B. Saunders Company, 2001, pp 1419-1432.
21. Charalambous, C, Paschalides, C, Sadiq, S, Tryfonides, M, Hirst, P, Paul, AS: Weight bearing following intra-articular steroid injection of the knee: survey of current practice and review of the available evidence. *Rheumatol Int,* 22**:** 185-187, 2002.
22. Liddell, WG, Carmichael, CR, McHugh, NJ: Joint and soft tissue injections: a survey of general practitioners. *Rheumatology (Oxford),* 44**:** 1043-1046, 2005.
23. Ekeberg, OM, Bautz-Holter, E, Tveita, EK, Juel, NG, Kvalheim, S, Brox, JI: Subacromial ultrasound guided or systemic steroid injection for rotator cuff disease: randomised double blind study. *BMJ,* 338**:** a3112, 2009.
24. Singh, JA, Saag, KG, Bridges, SL, Jr., Akl, EA, Bannuru, RR, Sullivan, MC, Vaysbrot, E, McNaughton, C, Osani, M, Shmerling, RH, Curtis, JR, Furst, DE, Parks, D, Kavanaugh, A, O'Dell, J, King, C, Leong, A, Matteson, EL, Schousboe, JT, Drevlow, B, Ginsberg, S, Grober, J, St Clair, EW, Tindall, E, Miller, AS, McAlindon, T, American College of, R: 2015 American College of Rheumatology Guideline for the Treatment of Rheumatoid Arthritis. *Arthritis Care Res (Hoboken),* 68**:** 1-25, 2016.
25. Brinks, A, van Rijn, RM, Willemsen, SP, Bohnen, AM, Verhaar, JA, Koes, BW, Bierma-Zeinstra, SM: Corticosteroid injections for greater trochanteric pain syndrome: a randomized controlled trial in primary care. *Ann Fam Med,* 9**:** 226-234, 2011.
26. Dorleijn, DMJ, Luijsterburg, PAJ, Reijman, M, Kloppenburg, M, Verhaar, JAN, Bindels, PJE, Bos, PK, Bierma-Zeinstra, SMA: Intramuscular glucocorticoid injection versus placebo injection in hip osteoarthritis: a 12-week blinded randomised controlled trial. *Ann Rheum Dis,* 77**:** 875-882, 2018.
27. Arroll, B, Goodyear-Smith, F: Corticosteroid injections for osteoarthritis of the knee: meta-analysis. *Bmj,* 328**:** 869, 2004.
28. Waterbrook, AL, Balcik, BJ, Goshinska, AJ: Blood Glucose Levels After Local Musculoskeletal Steroid Injections in Patients With Diabetes Mellitus: A Clinical Review. *Sports Health,* 9**:** 372-374, 2017.
29. Aleem, AW, Syed, UAM, Nicholson, T, Getz, CL, Namdari, S, Beredjiklian, PK, Abboud, JA: Blood Glucose Levels in Diabetic Patients Following Corticosteroid Injections into the Subacromial Space of the Shoulder. *Arch Bone Jt Surg,* 5**:** 315-321, 2017.
30. Richardson, SS, Schairer, WW, Sculco, TP, Sculco, PK: Comparison of Infection Risk with Corticosteroid or Hyaluronic Acid Injection Prior to Total Knee Arthroplasty. *J Bone Joint Surg Am,* 101**:** 112-118, 2019.
31. Werner, BC, Cancienne, JM, Browne, JA: The Timing of Total Hip Arthroplasty After Intraarticular Hip Injection Affects Postoperative Infection Risk. *J Arthroplasty,* 31**:** 820-823, 2016.
32. Altman, R, Asch, E, Bloch, D, Bole, G, Borenstein, D, Brandt, K, Christy, W, Cooke, TD, Greenwald, R, Hochberg, M, et al.: Development of criteria for the classification and reporting of osteoarthritis. Classification of osteoarthritis of the knee. Diagnostic and Therapeutic Criteria Committee of the American Rheumatism Association. *Arthritis Rheum,* 29**:** 1039-1049, 1986.

33. Emrani, PS, Katz, JN, Kessler, CL, Reichmann, WM, Wright, EA, McAlindon, TE, Losina, E: Joint space narrowing and Kellgren-Lawrence progression in knee osteoarthritis: an analytic literature synthesis. *Osteoarthritis Cartilage,* 16**:** 873-882, 2008.
34. Kellgren, JH, Lawrence, JS: Radiological assessment of osteo-arthrosis. *Ann Rheum Dis,* 16**:** 494-502, 1957.
35. Shah, A, Mak, D, Davies, AM, James, SL, Botchu, R: Musculoskeletal Corticosteroid Administration: Current Concepts. *Can Assoc Radiol J,* 70**:** 29-36, 2019.
36. Hermans, J, Bierma-Zeinstra, SM, Bos, PK, Verhaar, JA, Reijman, M: The most accurate approach for intra-articular needle placement in the knee joint: a systematic review. *Semin Arthritis Rheum,* 41**:** 106-115, 2011.
37. Mishra, P, Stringer, MD: Sciatic nerve injury from intramuscular injection: a persistent and global problem. *Int J Clin Pract,* 64**:** 1573-1579, 2010.
38. Jung Kim, H, Hyun Park, S: Sciatic nerve injection injury. *J Int Med Res,* 42**:** 887-897, 2014.
39. Larkin, TA, Ashcroft, E, Hickey, BA, Elgellaie, A: Influence of gender, BMI and body shape on theoretical injection outcome at the ventrogluteal and dorsogluteal sites. *J Clin Nurs,* 27**:** e242-e250, 2018.
40. Reijman, M, Pols, HA, Bergink, AP, Hazes, JM, Belo, JN, Lievense, AM, Bierma-Zeinstra, SM: Body mass index associated with onset and progression of osteoarthritis of the knee but not of the hip: the Rotterdam Study. *Ann Rheum Dis,* 66**:** 158-162, 2007.
41. Rienstra, W, Blikman, T, Mensink, FB, van Raay, JJ, Dijkstra, B, Bulstra, SK, Stevens, M, van den Akker-Scheek, I: The Modified painDETECT Questionnaire for Patients with Hip or Knee Osteoarthritis: Translation into Dutch, Cross-Cultural Adaptation and Reliability Assessment. *PLoS One,* 10**:** e0146117, 2015.
42. Craig, CL, Marshall, AL, Sjostrom, M, Bauman, AE, Booth, ML, Ainsworth, BE, Pratt, M, Ekelund, U, Yngve, A, Sallis, JF, Oja, P: International physical activity questionnaire: 12-country reliability and validity. *Med Sci Sports Exerc,* 35**:** 1381-1395, 2003.
43. Maillefert, JF, Kloppenburg, M, Fernandes, L, Punzi, L, Gunther, KP, Martin Mola, E, Lohmander, LS, Pavelka, K, Lopez-Olivo, MA, Dougados, M, Hawker, GA: Multi-language translation and cross-cultural adaptation of the OARSI/OMERACT measure of intermittent and constant osteoarthritis pain (ICOAP). *Osteoarthritis Cartilage,* 17**:** 1293-1296, 2009.
44. Herdman, M, Gudex, C, Lloyd, A, Janssen, M, Kind, P, Parkin, D, Bonsel, G, Badia, X: Development and preliminary testing of the new five-level version of EQ-5D (EQ-5D-5L). *Qual Life Res,* 20**:** 1727-1736, 2011.
45. Versteegh, M, Vermeulen, K, Evers, S, de Wit, G, Prenger, R, Stolk, E: Dutch Tariff for the Five-Level Version of EQ-5D. *Value Health,* 19**:** 343-352, 2016.
46. de Groot, IB, Favejee, MM, Reijman, M, Verhaar, JA, Terwee, CB: The Dutch version of the Knee Injury and Osteoarthritis Outcome Score: a validation study. *Health Qual Life Outcomes,* 6**:** 16, 2008.
47. Pham, T, van der Heijde, D, Altman, RD, Anderson, JJ, Bellamy, N, Hochberg, M, Simon, L, Strand, V, Woodworth, T, Dougados, M: OMERACT-OARSI initiative: Osteoarthritis Research Society International set of responder criteria for osteoarthritis clinical trials revisited. *Osteoarthritis Cartilage,* 12**:** 389-399, 2004.
48. iMCQ: *i*Medical Consumption Questionnaire. http://wwwimtanl/questionnaires, Assessed October 2016.
49. Henriksen, M, Christensen, R, Klokker, L, Bartholdy, C, Bandak, E, Ellegaard, K, Boesen, MP, Riis, RG, Bartels, EM, Bliddal, H: Evaluation of the benefit of corticosteroid injection before exercise therapy in patients with osteoarthritis of the knee: a randomized clinical trial. *JAMA Intern Med,* 175**:** 923-930, 2015.

50. KOOS: Knee injury and Osteoarthritis Outcome Score (KOOS) questionnaire. Assessed October 2016.
51. Mills, KA, Naylor, JM, Eyles, JP, Roos, EM, Hunter, DJ: Examining the Minimal Important Difference of Patient-reported Outcome Measures for Individuals with Knee Osteoarthritis: A Model Using the Knee Injury and Osteoarthritis Outcome Score. *J Rheumatol,* 43**:** 395-404, 2016.
52. Altman, DG: Comparability of Randomized Groups. *J R Stat Soc Ser D-Stat,* 34**:** 125-136, 1985.
53. de Boer, MR, Waterlander, WE, Kuijper, LD, Steenhuis, IH, Twisk, JW: Testing for baseline differences in randomized controlled trials: an unhealthy research behavior that is hard to eradicate. *Int J Behav Nutr Phys Act,* 12**:** 4, 2015.
54. Rehal, S, Morris, TP, Fielding, K, Carpenter, JR, Phillips, PP: Non-inferiority trials: are they inferior? A systematic review of reporting in major medical journals. *BMJ Open,* 6**:** e012594, 2016.
55. Bellamy, N, Buchanan, WW, Goldsmith, CH, Campbell, J, Stitt, LW: Validation study of WOMAC: a health status instrument for measuring clinically important patient relevant outcomes to antirheumatic drug therapy in patients with osteoarthritis of the hip or knee. *J Rheumatol,* 15**:** 1833-1840, 1988.
56. van Middelkoop, M, Arden, NK, Atchia, I, Birrell, F, Chao, J, Rezende, MU, Lambert, RG, Ravaud, P, Bijlsma, JW, Doherty, M, Dziedzic, KS, Lohmander, LS, McAlindon, TE, Zhang, W, Bierma-Zeinstra, SM: The OA Trial Bank: meta-analysis of individual patient data from knee and hip osteoarthritis trials show that patients with severe pain exhibit greater benefit from intra-articular glucocorticoids. *Osteoarthritis Cartilage,* 24**:** 1143-1152, 2016.

Table 1. Eligibility criteria

Inclusion criteria:

1) contacted their GP* (consultation and/or repeat pain medication prescription) due to knee OA** (ICPC† L90 or L15) during the past five years;
2) aged 45 years and over;
3) symptomatic knee OA for at least 3 months prior to enrolment;
4) a minimum score of 3 on the NRS# asking about the severity of knee pain averaged over the past week (0-10; 0=no knee pain);
5) glucocorticoid injection is indicated in this patient

Exclusion criteria:

1) use of oral glucocorticoids;
2) intra-articular injection in a knee in the previous 6 months;
3) allergy to glucocorticoids;
4) local or systemic infection, recent vaccination with live attenuated vaccine;
5) type 1 diabetes mellitus, type 2 diabetes mellitus on insulin therapy, poorly controlled type 2 diabetes mellitus;
6) presence of inflammatory rheumatic diseases (such as rheumatoid arthritis, psoriatic arthritis, spondylartropathies);
7) coagulopathy, use of anticoagulants, use of dual antiplatelet therapy;
8) a history of gastric/duodenal ulcer or a present gastric/duodenal ulcer;
9) currently receiving care of an orthopaedic surgeon for OA of the hip and/or knee;
10) incapacity to complete questionnaires in Dutch;
11) incapacity to give informed consent.

GP General Practitioner ***OA Osteoarthritis* *†ICPC International Classification of Primary Care # Numeric Rating Scale*

Figure 1. Flow-chart of patient selection

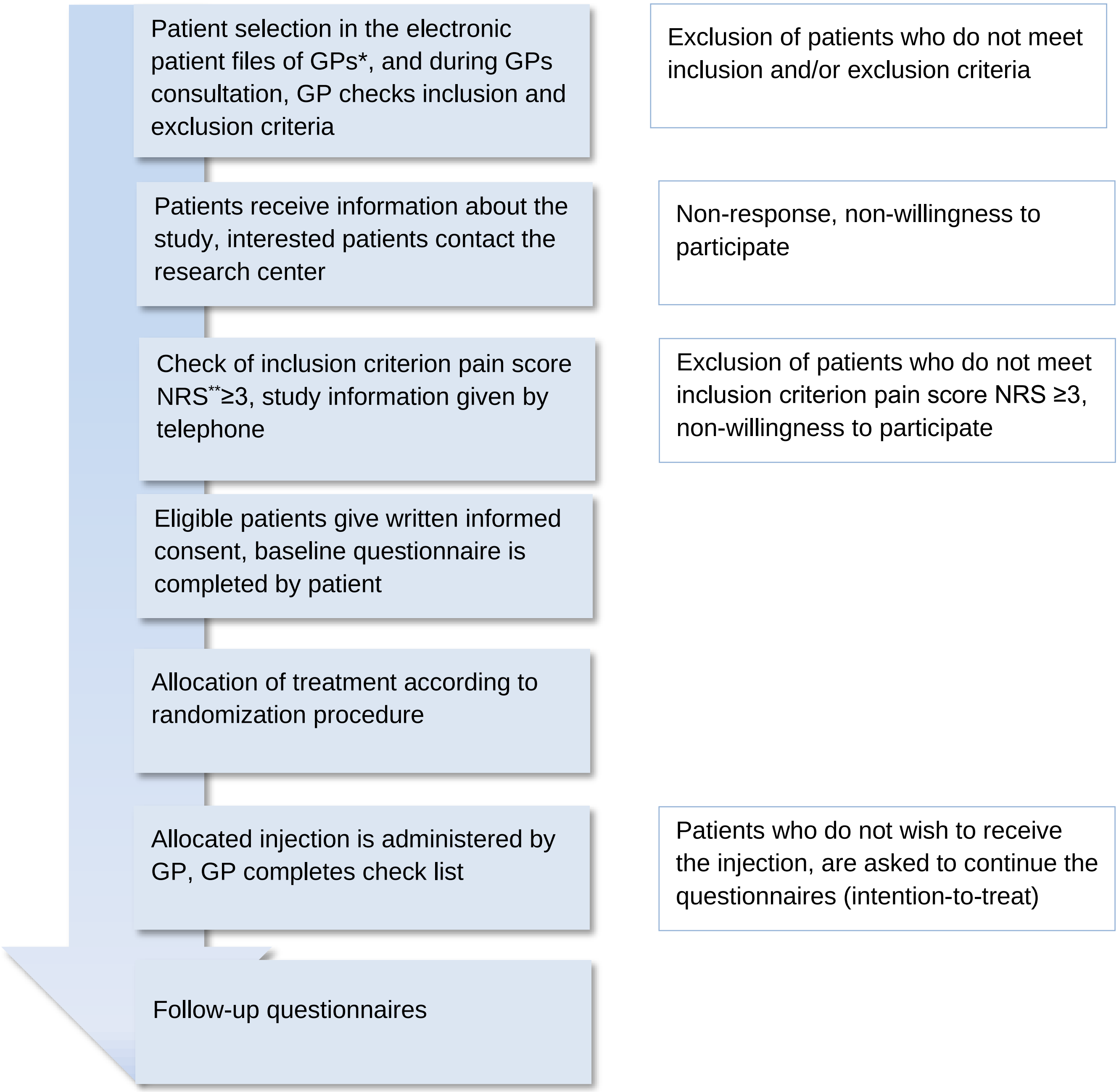

**GP General Practitioner   ** Numeric Rating Scale*

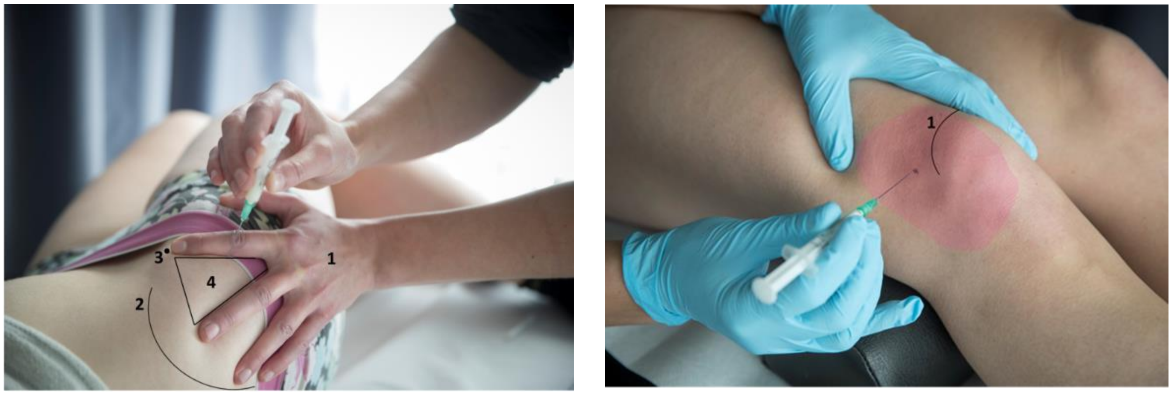

A.
1: palm of hand on greater trochanter
2: iliac crest
3: anterior superior iliac spine
4: area for injection

B.
1: upper border of the patella

Figure 2. ventrogluteal intramuscular and superolateral intra-articular injection techniques

Table 2: scheduled measurements of primary and secondary outcomes

Measurement	Baseline	Injection	2 weeks fu	4 weeks fu	8 weeks fu	12 weeks fu	24 weeks fu
Primary outcome measure							
KOOS pain subscale	x		x	x	x	x	x
Secondary outcome measures							
Adverse events			x				
Hospitalization			x	x	x	x	x
Co-interventions (iMCQ)			x	x	x	x	x
Medication use for knee OA	x		x	x	x	x	x
Re-injection with glucocorticoid					x	x	x
KOOS stiffness	x		x	x	x	x	x
KOOS function in daily living	x		x	x	x	x	x
KOOS sports and recreation	x		x	x	x	x	x
KOOS QoL	x		x	x	x	x	x
ICOAP	x		x	x	x	x	x
OMERACT OARSI responder criteria			x	x	x	x	x
Knee pain over past week (NRS)	x	x	x	x	x	x	x
Perceived recovery (Likert scale)			x	x	x	x	x
Knee complaint characteristics	x		x	x	x	x	x
Health related QoL	x						
Additional measurements							
Radiograph of study knee						x	
Check of ACR criteria		x					
Painfulness of injection (NRS)			x				
Demographic information	x						
Co-morbidity	x						
Physical activity over the past week (IPAQ short)	x						
Neuropathic pain (Modified painDETECT Questionnaire)	x						
Patients' preferred injection site	x						
Patients' expected treatment response	x						

Table 3: AIC of the tested covariance type.

**Covariance type**	**AIC**
Diagonal	7241.5
AR(1)	6723.1
AR(1): Heterogeneous	6703.5
ARMA(1,1)	6689.2
Compound Symmetry	6758.4
Compound Symmetry: Correlation Metric	6758.3
Compound Symmetry: Heterogeneous	6759.9
Factor Analytic: First Order	6739.2
Factor Analytic: First Order, Heterogeneous	6898.2
Huynh-Feldt	6806.9
Scaled Identity	7238.6
Toeplitz	6688.1
Toeplitz: Heterogeneous	6688.7
Unstructured	6654.1
Unstructured: Correlation Metric	6654.2
