# Supplementary material for: Effect of Intramuscular vs Intra-articular Glucocorticoid Injection on Pain Among Adults With Knee Osteoarthritis: The KIS Randomized Clinical Trial
Source: JAMA Netw Open. 2022 Apr 5;5(4):e224852. doi: 10.1001/jamanetworkopen.2022.4852 (PMC8984774; doi:10.1001/jamanetworkopen.2022.4852)
Supplement: Supplement 2. — eTable 1. Results of the Linear Mixed Models With Repeated Measurements for Between-Group Differences Regarding Secondary Outcomes Based on an Intention-to-Treat Analysis eTable 2. Results of Generalized Estimating Equations With Repeated Measurements for Intergroup Differences Regarding Responders and Perceived Recovery Based on an Intention-to-Treat Analysis eTable 3. Adverse Events at 2 Weeks’ Follow-up eTable 4. Cointerventions of Patients in the Two Groups eFigure. Unadjusted KOOS Pain Scores of Patients Included in Per-Protocol Analysis Over the Entire Follow-up [file jamanetwopen-e224852-s002.pdf]

Supplementary Online Content

Wang Q, Mol MF, Bos PK, et al. Effect of intramuscular vs intra-articular glucocorticoid injection on pain among adults with knee osteoarthritis: the KIS randomized clinical trial. *JAMA Netw Open*. 2022;5(3):e224852. doi:10.1001/jamanetworkopen.2022.4852

eTable 1. Results of the Linear Mixed Models With Repeated Measurements for Between-Group Differences Regarding Secondary Outcomes Based on an Intention-to-Treat Analysis

eTable 2. Results of Generalized Estimating Equations With Repeated Measurements for Intergroup Differences Regarding Responders and Perceived Recovery Based on an Intention-to-Treat Analysis

eTable 3. Adverse Events at 2 Weeks' Follow-up

eTable 4. Cointerventions of Patients in the Two Groups

eFigure. Unadjusted KOOS Pain Scores of Patients Included in Per-Protocol Analysis Over the Entire Follow-up

This supplementary material has been provided by the authors to give readers additional information about their work.

© 2022 Wang Q et al. *JAMA Network Open.*

eTable 1. Results of the linear mixed models with repeated measurements for between-group differences regarding secondary outcomes based on an intention-to-treat analysis.

	IM (N=74) Mean (SD)*	IA (N=71) Mean (SD)*	Mean difference (95% CI)#	P values§	Effect size
**KOOS symptom**					
2 weeks	63.9 (15.4)	69.2 (17.1)	-3.7 (-9.4 to 1.9)	0.19	-0.23
4 weeks	65.4 (17.4)	70.9 (17.8)	-3.7 (-9.8 to 2.4)	0.23	-0.21
8 weeks	67.5 (18.0)	66.3 (18.0)	0.8 (-5.6 to 7.3)	0.80	0.04
12 weeks	64.3 (19.0)	65.9 (19.8)	-0.9 (-7.5 to 5.7)	0.78	-0.05
24 weeks	63.5 (20.2)	63.8 (20.2)	0.9 (-6.0 to 7.9)	0.79	0.04
**KOOS function**					
2 weeks	62.3 (21.1)	67.8 (21.6)	-2.8 (-10.0 to 4.4)	0.44	-0.13
4 weeks	63.2 (21.4)	68.4 (20.0)	-2.9 (-9.9 to 4.2)	0.42	-0.14
8 weeks	66.9 (21.6)	65.4 (21.9)	2.0 (-5.5 to 9.5)	0.60	0.09
12 weeks	62.6 (22.3)	64.9 (20.9)	-0.4 (-7.7 to 7.0)	0.92	-0.02
24 weeks	60.1 (22.8)	60.8 (22.7)	2.0 (-5.7 to 9.7)	0.61	0.09
**KOOS sport & recreation**					
2 weeks	25.9 (22.5)	29.1 (27.0)	-2.0 (-10.5 to 6.6)	0.65	-0.08
4 weeks	25.7 (22.4)	32.0 (26.8)	-5.0 (-13.4 to 3.4)	0.24	-0.20

© 2022 Wang Q et al. *JAMA Network Open.*

8 weeks	30.1 (23.2)	28.5 (24.5)	2.4 (-5.7 to 10.6)	0.55	0.10
12 weeks	29.3 (25.0)	26.7 (25.9)	3.0 (-5.7 to 11.7)	0.50	0.12
24 weeks	23.1 (22.3)	24.8 (27.8)	-0.3 (-8.9 to 8.4)	0.95	-0.01
**KOOS QoL**					
2 weeks	39.3 (17.3)	42.9 (20.2)	-0.2 (-6.6 to 6.1)	0.95	-0.01
4 weeks	39.9 (19.3)	46.0 (20.8)	-2.4 (-9.2 to 4.3)	0.48	-0.12
8 weeks	43.9 (19.8)	45.4 (21.2)	0.9 (-6.0 to 7.8)	0.80	0.04
12 weeks	41.5 (20.6)	45.9 (21.3)	-1.7 (-8.6 to 5.3)	0.64	-0.08
24 weeks	38.8 (20.4)	41.6 (19.9)	0.04 (-6.7 to 6.7)	0.99	0.002
**WOMAC pain**					
2 weeks	36.1 (21.1)	30.2 (21.5)	3.9 (-3.5 to 11.2)	0.30	0.18
4 weeks	34.6 (21.1)	29.4 (20.0)	3.7 (-3.3 to 10.8)	0.30	0.18
8 weeks	31.1 (20.8)	32.1 (20.0)	-1.0 (-8.2 to 6.1)	0.78	-0.05
12 weeks	35.4 (21.5)	32.8 (22.1)	1.6 (-5.9 to 9.0)	0.68	0.07
24 weeks	38.2 (22.4)	37.4 (22.1)	-0.8 (-8.4 to 6.9)	0.84	-0.04
**WOMAC function**					
2 weeks	37.7 (21.1)	32.2 (21.6)	2.8 (-4.4 to 10.0)	0.44	0.13
4 weeks	36.8 (21.4)	31.6 (20.0)	2.9 (-4.2 to 10.0)	0.42	0.14
8 weeks	33.1 (21.6)	34.6 (21.9)	-2.0 (-9.5 to 5.5)	0.60	-0.09

© 2022 Wang Q et al. *JAMA Network Open.*

12 weeks	37.4 (22.3)	35.1 (20.9)	0.4 (-7.0 to 7.7)	0.92	0.02
24 weeks	39.9 (22.8)	39.2 (22.7)	-2.0 (-9.7 to 5.7)	0.61	-0.09
**WOMAC stiffness**					
2 weeks	45.2 (18.8)	40.5 (22.3)	3.6 (-3.6 to 10.8)	0.32	0.17
4 weeks	42.4 (22.1)	37.3 (23.5)	4.0 (-3.9 to 11.8)	0.32	0.17
8 weeks	40.1 (24.0)	43.1 (23.2)	-2.4 (-10.7 to 5.8)	0.56	-0.10
12 weeks	44.2 (24.0)	43.9 (23.7)	0.1 (-8.0 to 8.3)	0.98	0.004
24 weeks	45.3 (24.2)	48.2 (23.9)	-3.9 (-12.1 to 4.2)	0.34	-0.16
**WOMAC total**					
2 weeks	38.0 (20.2)	32.5 (20.8)	3.1 (-3.8 to 10.1)	0.38	-0.15
4 weeks	36.8 (20.6)	31.6 (19.4)	3.2 (-3.6 to 10.1)	0.35	-0.16
8 weeks	33.2 (20.7)	34.8 (20.9)	-1.7 (-9.0 to 5.5)	0.63	0.08
12 weeks	37.6 (21.5)	35.4 (20.4)	0.7 (-6.4 to 7.8)	0.85	-0.03
24 weeks	40.0 (22.0)	40.0 (21.9)	-1.9 (-9.3 to 5.6)	0.62	0.09
**NRS pain (0-10)**					
2 weeks	4.9 (2.2)	4.3 (2.6)	0.5 (-0.3 to 1.3)	0.22	0.21
4 weeks	5.0 (2.3)	4.1 (2.3)	0.8 (0.02 to 1.6)	0.05	0.34
8 weeks	4.5 (2.2)	5.0 (2.4)	-0.4 (-1.2 to 0.4)	0.29	-0.17
12 weeks	5.2 (2.2)	4.8 (2.5)	0.4 (-0.4 to 1.2)	0.36	0.17

© 2022 Wang Q et al. *JAMA Network Open.*

24 weeks	5.5 (2.2)	5.5 (2.3)	0.02 (-0.8 to 0.8)	0.96	0.01
**ICOAP constant pain score**					
2 weeks	30.4 (22.1)	23.2 (22.3)	5.7 (-2.0 to 13.4)	0.14	0.25
4 weeks	31.4 (23.3)	25.2 (21.2)	4.9 (-2.8 to 12.7)	0.21	0.22
8 weeks	28.4 (23.1)	28.8 (22.2)	0.1 (-8.0 to 8.2)	0.98	0.004
12 weeks	33.0 (22.4)	28.7 (22.6)	4.6 (-3.3 to 12.6)	0.25	0.20
24 weeks	33.3 (22.7)	32.0 (22.7)	0.8 (-7.3 to 8.9)	0.84	0.03
**ICOAP intermittent pain score**					
2 weeks	34.9 (21.4)	26.1 (21.6)	6.6 (-0.9 to 14.1)	0.08	0.30
4 weeks	36.4 (20.8)	29.5 (21.5)	5.0 (-2.3 to 12.3)	0.18	0.23
8 weeks	33.4 (21.9)	32.4 (20.2)	0.5 (-6.9 to 8.0)	0.88	0.02
12 weeks	35.9 (22.4)	32.2 (23.9)	3.2 (-4.9 to 11.2)	0.44	0.14
24 weeks	38.6 (20.5)	35.3 (22.8)	2.1 (-5.6 to 9.7)	0.59	0.10
**ICOAP total score**					
2 weeks	32.9 (21.0)	24.8 (21.3)	6.3 (-1.1 to 13.6)	0.09	0.29
4 weeks	34.1 (21.2)	27.6 (20.8)	5.0 (-2.3 to 12.4)	0.18	0.24
8 weeks	31.1 (21.8)	30.7 (20.6)	0.4 (-7.2 to 7.9)	0.92	0.02
12 weeks	34.6 (21.5)	30.6 (22.9)	3.9 (-3.9 to 11.7)	0.32	0.18
24 weeks	36.2 (20.5)	33.8 (22.2)	1.5 (-6.0 to 9.1)	0.69	0.07

© 2022 Wang Q et al. *JAMA Network Open.*

**EQ-5D-5L (-0.446 to 1)**					
4 weeks	0.67 (0.26)	0.75 (0.21)	-0.04 (-0.13 to 0.04)	0.30	-0.17
24 weeks	0.66 (0.27)	0.69 (0.25)	0.01 (-0.08 to 0.10)	0.81	0.04

IM, intramuscular injection; IA, intra-articular injection; SD, standard deviation; CI, confidence interval; KOOS, Knee injury and Osteoarthritis Outcome Score (0 indicates extreme symptoms); QOL, quality of life; WOMAC, Western Ontario and McMaster Universities Osteoarthritis Index (0 indicates no symptom); NRS, numeric rating scale (0 indicates no pain); ICOAP, intermittent and constant osteoarthritis pain (0 indicates no pain); EQ-5D-5L, Euroqol with 5 dimensions and 5 response levels (-0.446 indicates worse health related QoL).

Scores were scaled with a range of 0 to 100, or otherwise stated. There was no missing value in the model covariates, all the patients (145) were included in modelling.

* Observed means and SDs, unadjusted values.

IA injection as reference, adjusted for baseline score, sex, presence of depression and duration of knee OA. Calculated based on estimated marginal percentages.

§ P values for superiority tests.

© 2022 Wang Q et al. *JAMA Network Open.*

eTable 2. Results of generalized estimating equations with repeated measurements for intergroup differences regarding responders and perceived recovery based on an intention-to-treat analysis.

	IM injection N (%)*	IA injection N (%)*	OR (95% CI)#	P values§
**OMERACT OARSI responder criteria**				
2 weeks	35/70 (50)	43/67 (64)	0.8 (0.4 to 1.6)	0.490
4 weeks	35/71 (49)	47/69 (68)	0.6 (0.3 to 1.2)	0.119
8 weeks	38/67 (57)	38/69 (55)	1.4 (0.7 to 2.8)	0.360
12 weeks	29/69 (42)	37/68 (54)	0.8 (0.4 to 1.7)	0.550
24 weeks	20/69 (29)	33/68 (48)	0.5 (0.2 to 1.1)	0.107
**Perceived recovery**				
2 weeks	43/70 (61)	52/67 (78)	0.6 (0.3 to 1.2)	0.158
4 weeks	45/71 (63)	53/69 (77)	0.6 (0.3 to 1.3)	0.191
8 weeks	42/67 (63)	43/69 (62)	1.1 (0.5 to 2.2)	0.842
12 weeks	32/69 (46)	39/68 (57)	0.7 (0.3 to 1.4)	0.314
24 weeks	28/69 (41)	31/68 (46)	0.9 (0.5 to 1.8)	0.756

IM, intramuscular injection; IA, intra-articular injection; OR, odds ratio; CI, confidence interval. There was no missing value in the model covariates, all the patients (145) were included in modelling.

* Observed and unadjusted values.

IA injection as reference, Adjusted for baseline KOOS pain score, sex, presence of depression and duration of knee OA.

§ P values for superiority tests.

© 2022 Wang Q et al. *JAMA Network Open.*

eTable 3. Adverse events at 2 weeks' follow-up.

	**IM (N=72)**<br>**N (%)**	**IA (N=66)**<br>**N (%)**
**Hot flush**	7 (10)	14 (21)
**Headache**	10 (14)	12 (18)
**Irregular menstruation**	1 (1)	0
**Allergic reaction**		
Itching	2 (3)	5 (8)
Dyspnea	2 (3)	1 (1)
**Pain in extremity**	0	2 (3)
**Cramps in extremity**	1 (1)	0
**Sweating**	1 (1)	1 (1)
**Fatigue**	1 (1)	0
**Hypertension**	0	1 (1)
**Palpitation**	1 (1)	1 (1)
**Hyperglycaemia**	0	1 (1)
**Nervous**	1 (1)	0

IM, intramuscular injection; IA, intra-articular injection.

© 2022 Wang Q et al. *JAMA Network Open.*

eTable 4. Cointerventions of patients in the two groups.

	Follow-ups	IM (N=72) N (%)	IA (N=66) N (%)
**Pharmacological co-interventions**			
Acetaminophen	Baseline	16 (22)	23 (35)
	2	10 (14)	9 (14)
	4	9 (13)	11 (17)
	8	11 (15)	14 (21)
	12	11 (15)	14 (21)
	24	12 (17)	14 (21)
NSAIDs	Baseline	14 (19)	11 (17)
	2	8 (11)	7 (11)
	4	9 (13)	7 (11)
	8	13 (18)	6 (10)
	12	10 (14)	5 (8)
	24	12 (17)	5 (8)
Opiates (Tramadol)¶	Baseline	2 (3)	2 (3)
	2	2 (3)	0
	4	1 (1)	0
	8	1 (1)	0
	12	1 (1)	0
	24	2 (3)	0
Additional injection (IA glucocorticoids injection)	8	0	4 (6)
	12	2 (3)	1 (2)
	24	2 (3)	4 (6)
**Visited medical care provider for knee osteoarthritis**			
Primary care	2	5 (7)	1 (2)
	4	0	3 (5)
	8	4 (6)	4 (6)
	12	4 (6)	3 (5)
	24	12 (17)	9 (14)
Secondary care	2	0	1 (2)
	4	0	0

© 2022 Wang Q et al. *JAMA Network Open.*

	8	0	1 (2)
	12	1 (1)	3 (5)
	24	2 (3)	4 (6)
Physical therapist	2	6 (8)	2 (3)
	4	5 (7)	4 (6)
	8	5 (7)	3 (5)
	12	6 (8)	2 (3)
	24	9 (13)	3 (5)

IM, intramuscular injection; IA, intra-articular injection.

¶ Patients reported were all Tramadol; none of other types of opiates was reported.

© 2022 Wang Q et al. *JAMA Network Open.*

eFigure. Unadjusted KOOS pain scores of patients included in per-protocol analysis over the entire follow-up. Point estimates indicate mean scores; error bars indicate 95% confidence intervals.

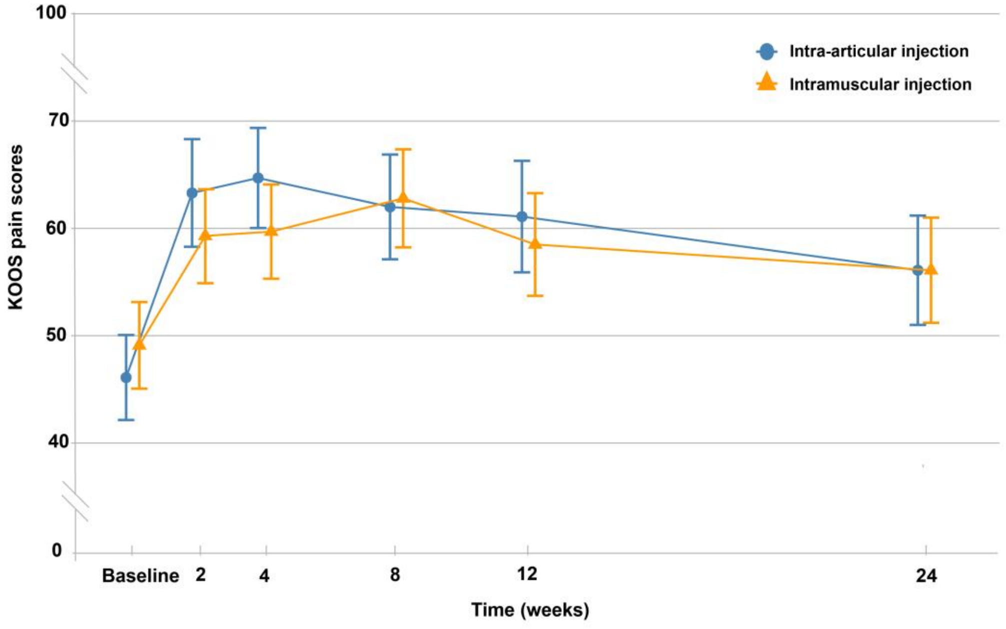

© 2022 Wang Q et al. *JAMA Network Open.*
