# Supplementary material for: Effect of Intramuscular vs Intra-articular Glucocorticoid Injection on Pain Among Adults With Knee Osteoarthritis: The KIS Randomized Clinical Trial
Source: JAMA Netw Open. 2022 Apr 5;5(4):e224852. doi: 10.1001/jamanetworkopen.2022.4852 (PMC8984774; doi:10.1001/jamanetworkopen.2022.4852)
Supplement: Supplement 3. — Data Sharing Statement [file jamanetwopen-e224852-s003.pdf]

Data Sharing Statement

Wang. Effect of Intramuscular vs Intra-articular Glucocorticoid Injection on Pain Among Adults With Knee Osteoarthritis. *JAMA Netw Open*. Published April 05, 2022.
doi:10.1001/jamanetworkopen.2022.4852

Data
Data available: Yes
Data types: Deidentified participant data
How to access data: Individual data will be shared by contacting the corresponding author on reasonable request.
When available: With publication

Supporting Documents
Document types: None

Additional Information
Who can access the data: Researchers whose proposed use of the data has been approved
Types of analyses: For a specified purpose
Mechanisms of data availability: after approval of a proposal
